# Factor Score Regression With Social Relations Model Components: A Case Study Exploring Antecedents and Consequences of Perceived Support in Families

**DOI:** 10.3389/fpsyg.2018.01699

**Published:** 2018-09-19

**Authors:** Justine Loncke, Veroni I. Eichelsheim, Susan J. T. Branje, Ann Buysse, Wim H. J. Meeus, Tom Loeys

**Affiliations:** ^1^Department of Data Analysis, Ghent University, Ghent, Belgium; ^2^Netherlands Institute for the Study of Crime and Law Enforcement, Amsterdam, Netherlands; ^3^Department of Youth and Family, Faculty of Social Sciences, Utrecht University, Amsterdam, Netherlands; ^4^Department of Experimental Clinical and Health Psychology, Ghent University, Ghent, Belgium

**Keywords:** factor score regression, family social relations model, perceived support, structural equation modeling, missing data

## Abstract

The family social relations model (SRM) is applied to identify the sources of variance in interpersonal dispositions in families, but the antecedents or consequences of those sources are rarely investigated. Simultaneous modeling of the SRM with antecedents or consequences using structural equation modeling (SEM) allows to do so, but may become computationally prohibitive in small samples. We therefore consider two factor score regression (FSR) methods: regression and Bartlett FSR. Based on full information maximum likelihood (FIML), we derive closed-form expressions for the regression and Bartlett factor scores in the presence of missingness. A simulation study in both a complete- and incomplete-case setting compares the performance of these FSR methods with SEM and an ANOVA-based approach. In both settings, the regression FIML factor scores as explanatory variable produces unbiased estimators with precision comparable to the SEM-estimators. When SRM-effects are used as dependent variables, none of the FSR methods are a suitable alternative for SEM. The latter result deviates from previous studies on FSR in more simple settings. As an example, we explore whether gender and past victimhood of relational and physical aggression are antecedents for family dynamics of perceived support, and whether those dynamics predict physical and relational aggression.

## 1. Introduction

Families can be seen as dynamic systems where the family members form interdependent subsystems (Cox and Paley, 2003). In such view the individual level, the dyadic and the family level play an important role. By adopting a round robin design, in which each family member rates a psychological concept concerning their relationship with other family members, a systematic investigation of those levels is possible.

The social relations model [SRM; (Kenny and La Voie, 1984)] with roles is a popular model in family research that allows to disentangle these complex family dynamics from round-robin data. According to the SRM, each dyadic measurement is potentially a function of four latent systematic sources of variance: an actor effect, a partner effect, a relationship effect, and a family effect. The actor effect describes a person's general level of response across partners. The partner effect describes the way family members generally behave toward a particular member and the relationship effect describes the unique adaptation of one person toward another, while controlling for both actor and partner effects. Lastly, the family effect reflects the average level of the dyadic measurement across all dyads in the family. The revelation of how those SRM-effects are associated with individual, relational, and family characteristics or outcomes could be an important next step for family researchers. However, SRM-effects are rarely being connected with possible antecedents or consequences (Cook, 1993).

A literature search on case studies exploring antecedents and consequences of SRM-effects revealed two different approaches. The first approach is a two-step approach, where first the SRM-effects are calculated using the raw data from the round robin matrix in an ANOVA-like manner (Kenny et al., 2006). Afterwards, these calculated SRM-effects or ANOVA scores are used to map the relationship with possible antecedents and/or consequences. Due to its simplicity, it is the foremost used approach in family research (e.g., Srivastava et al., 2010; Betts et al., 2011; Denissen et al., 2011; Shea, 2011). However, it is well known that sum score approaches, such as the ANOVA-based approach, suffer from severe limitations in terms of bias and missing data (Croon and van Veldhoven, 2007; Lüdtke et al., 2018). The second approach uses structural equation modeling (SEM), where the SRM-effects and their relations with their possible antecedents and/or consequences are estimated simultaneously (e.g., Branje et al., 2005; Delsing et al., 2005; Migerode et al., 2012). From a theoretical perspective, SEM is perfectly suitable to perform these analyses, since it simultaneously performs an estimation of the measurement and structural part. Due to this simultaneous estimation, SEM is a more complex model whereby researchers often run into problems and convergency issues. They might for example obtain improper solutions (i.e., Heywood cases) when SEM is applied to a small sample size or they might obtain biased estimates when parts of a complex model are misspecified (Hoshino and Bentler, 2011).

To circumvent these problems researchers have proposed the factor score regression (FSR) method, where the measurement and structural part are subsequently estimated (Hoshino and Bentler, 2011). FSR breaks the SEM method into three steps: (1) estimation of the parameters of the measurement model, (2) estimation of the factor scores, and (3) the path analysis or regression analysis. FSR does not only offer a possible solution to avoid problems with complex models, but is also perceived as much simpler by many practitioners. The performance of FSR methods has been studied previously in fairly simple settings (Tucker, 1971; Skrondal and Laake, 2001; Devlieger et al., 2016). Those studies indicate that FSR often turns out to be a valuable alternative. These findings should, however, be reevaluated for more complex models such as the SRM. Furthermore, most of these studies ignored the presence of missing values, a common issue in family studies with a round robin design.

We aim to fill that gap by studying the performance of several FSR methods for modeling relationships between SRM-effects and their possible antecedents and consequences in terms of bias, precision and type I error rate. The performance is explored in a complete-case setting as well as in the presence of missing data. Using simulation studies we investigate whether the good performance of the FSR methods in simple situations also holds for the more complex SRM.

Recently, Lüdtke et al. (2018) performed a similar study on the integration of covariates into the SRM, hereby relying on a plausible values approach. However, their study is substantially different from the one presented here. Their study focuses on the application of the SRM in groups without roles (for e.g., students within classes) while we are considering the family setting here (with a fixed group size equal to 4, and 4 distinct roles within each group). While most applications of the SRM without roles rely on an ANOVA-based approach (Schönbrodt et al., 2012), the SRM with roles is typically analyzed using Confirmatory Factor Analysis in the SEM-framework (Stas et al., 2015). As argued by several scholars (Gill and Swartz, 2001; Nestler, 2016) the SRM could be implemented in a multilevel modeling framework too, hereby relying on (Restricted) Maximum Likelihood (ML) Estimation. Because ML-estimation can quickly become intractable with a large amount of random effects (or latent factors), Lüdtke et al. (2013) proposed Bayesian methods for estimating the SRM (without roles) in the multilevel modeling framework. While their approach can be applied to the family SRM with roles as well, it is further complicated due to the role-specific SRM-effects requiring some constraints (see further), the higher number of random effects in the family setting, and the small group size. As noted by Lüdtke et al. (2018) the latter may often lead to biased estimates of SRM variances in the Bayesian framework. We therefore opted to pursue our investigations in the SEM-framework which also more naturally allows for measurement error.

As an illustrating example throughout this paper we will consider longitudinal data on perceived support in families. More specifically, we have approximately 500 four-member families (father, mother, target adolescent, and sibling) that participated in the RADAR-Y (Research on Adolescent Development and Relationships - Younger Cohort) study, from which we consider the first three waves. Previous studies of perceived support mainly focused on differences in perceived relational support between families, but it is also interesting to examine the relationship between the SRM-effects of perceived support and some external variables (Branje et al., 2005). First of all, we will investigate the consequences of the SRM-effects of perceived support. There is evidence that perceived support has a negative effect on adverse outcomes such as depression, problematic behavior and delinquency (Marcotte et al., 2002). Therefore, we will assess the impact of the SRM-effects of perceived support at wave 2 on physical and relational aggression of the target adolescent at wave 3. Furthermore, we will also map several antecedents of the SRM-effects of perceived support. Since Furman (1996) showed that girls tend to report more perceived social support than boys, we will check with the RADAR-Y data whether there is an effect of gender of the target adolescent on the SRM-effects of perceived support. Because social support tends to minimize distress associated with the experience of victimization (e.g., Holt and Espelage, 2007), we will also investigate whether perceived victimhood of physical and relational aggression at wave 1 can predict SRM-effects of perceived support at wave 2.

Our paper is organized as follows. We start with a description of the SRM and explain how it can disentangle dyadic measurements from a round robin design into meaningful components. Next, a detailed description is given of the RADAR-Y study, which is then used to illustrate the traditional SRM analysis and the popular ANOVA-based approach. Since the ANOVA-based approach suffers from severe limitations in terms of bias and missing data, we introduce FSR methods as an alternative. The performance of these FSR methods is compared with SEM and the ANOVA-based approach by means of a simulation study in a complete-case setting as well as in the presence of missing data. The best performing methods are illustrated using data on perceived support from the RADAR-Y study.

## 2. The family social relations model

The SRM decomposes dyadic measurements into four different effects at three different levels: the individual, the dyadic and the family level. Consider a dyadic measurement, *X*_*ij*_, where *i* describes the role of the rater and *j* the role of person being rated. In a four-person family *i* and *j* may be the father (F), mother (M), target adolescent (T) or sibling (S). In our illustration *X*_*TF*_, for example, represents the support the target adolescent perceives from the father. Self-ratings (i.e., *i* = *j*) are not considered here.

According to the SRM, each *X*_*ij*_ can be expressed as a linear function of a family effect (*Fam*), an actor effect (*Act*), a partner effect (*Par*), a relation-specific effect *Rel*, and measurement error (ϵ):

(1) $$\begin{array}{r} X_{ij}=Fam+Act_{i}+Par_{j}+Rel_{ij}+\epsilon_{ij} \end{array}$$

**Figure 1:**
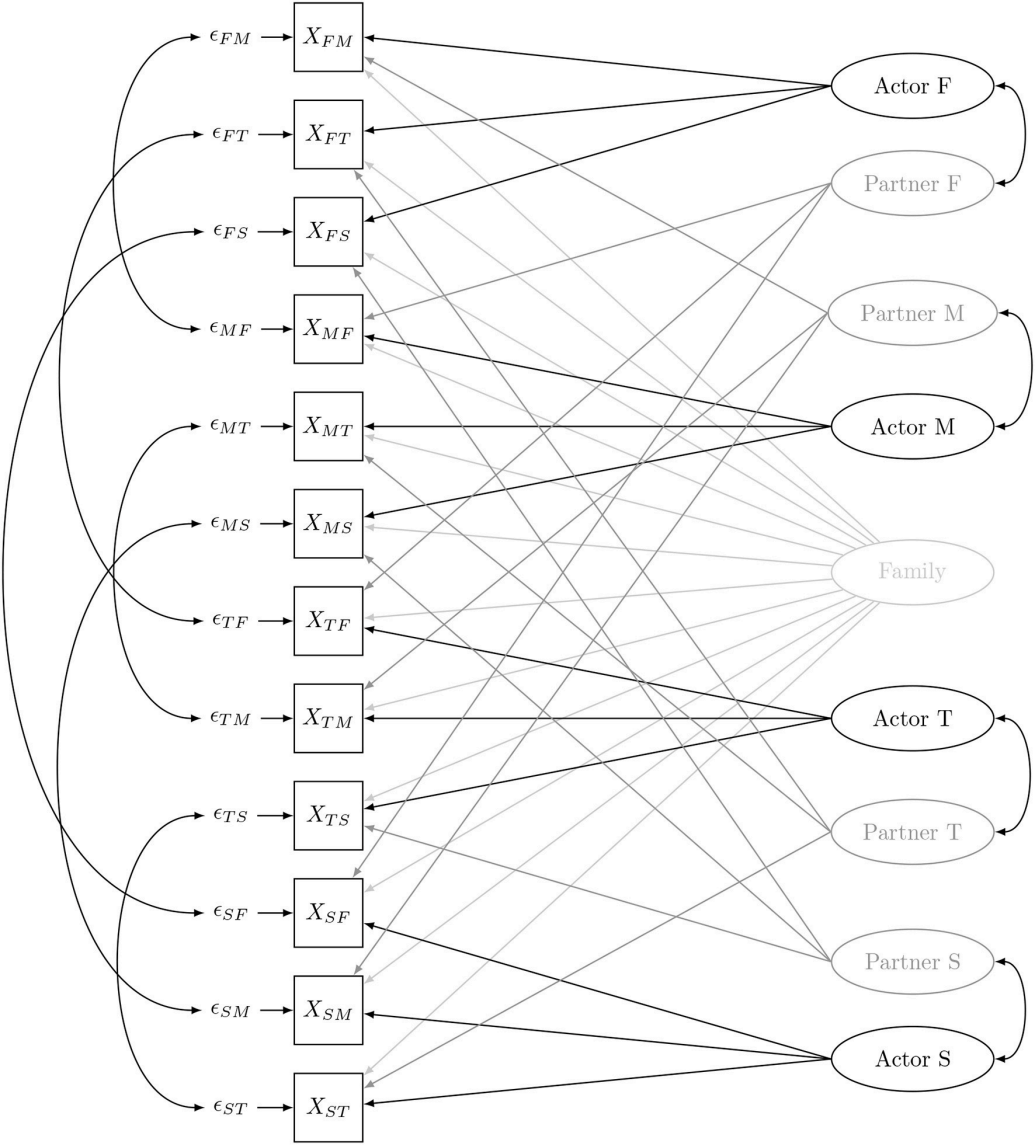
The family social relations model (*M*, mother; *F*, father; *T*, target adolescent; *S*, sibling).

Figure 1 shows the four-member model with the SRM-effects specified as latent variables and arrows pointing from those latent variables toward the dyadic measurements. Note, that when there is only one dyadic measurement per relationship, the relationship-specific effect *Rel*_*ij*_ cannot be disentangled from the measurement error ϵ_*ij*_. To separate the relationship effect from the measurement error at least two observations or indicators of each dyadic relation are needed (Back and Kenny, 2010). In the remainder of the paper, we will consider only one observation per dyadic measurement, and absorb the relationship effect in the error, since this approach is used in most SRM-applications.

In the confirmatory factor analysis (CFA) that is typically used for the SRM (Kenny et al., 2006), the paths from the SRM-effects to the dyadic measurements are fixed to one. Consequently, the SRM in Figure 1 has the following factor loading matrix Λ:

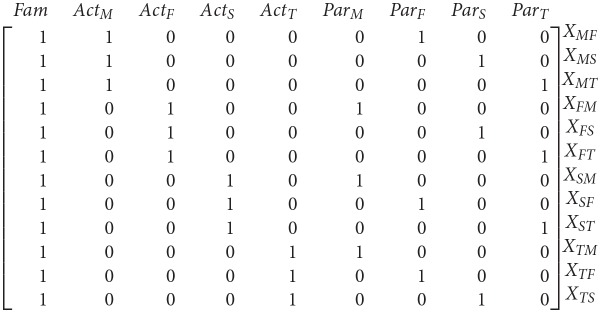



This factor loading matrix is not full rank, due to linear dependencies among the factor loadings of the latent variables. In specific the factor loadings of the family effect can be expressed in terms of the four actor or the four partner effects.

The SRM-components are considered to be possible sources of systematic variance in dyadic measurements across families. According to the SRM, the total variability in *X*_*ij*_ can be decomposed into the sum of the variances of the components, i.e.,

(2) $$\begin{array}{r} \text{Var}\left(X_{ij}\right)=\text{Var}\left(Fam\right)+\text{Var}\left(Act_{i}\right)+\text{Var}\left(Par_{j}\right)+\text{Var}\left(Rel_{ij}+\epsilon_{ij}\right) \end{array}$$

where the latter two are taken together in the case of only one observation per dyadic measurement. This decomposition can be done for all 12 dyadic measurements. Identifying the most important sources of variability is the primary aim of most SRM-studies.

Furthermore, SRM-effects are assumed to be independent, except for some that are related through patterns of reciprocity (Cook and Kenny, 2004), either at the individual level or at the relationship level. These reciprocities are indicated in Figure 1 by two-headed arrows. We refer to Cook and Kenny (2004) for a detailed discussion on their meaning. Consequently, the SRM has the following factor covariance matrix Ψ:

$$\Psi =\left[\begin{array}{ccccccccc} \sigma_{fam}^{2} & 0 & 0 & 0 & 0 & 0 & 0 & 0 & 0\\ 0 & \sigma_{Act_{M}}^{2} & 0 & 0 & 0 & \sigma_{M} & 0 & 0 & 0\\ 0 & 0 & \sigma_{Act_{F}}^{2} & 0 & 0 & 0 & \sigma_{T} & 0 & 0\\ 0 & 0 & 0 & \sigma_{Act_{S}}^{2} & 0 & 0 & 0 & \sigma_{S} & 0\\ 0 & 0 & 0 & 0 & \sigma_{Act_{T}}^{2} & 0 & 0 & 0 & \sigma_{T}\\ 0 & \sigma_{M} & 0 & 0 & 0 & \sigma_{Par_{M}}^{2} & 0 & 0 & 0\\ 0 & 0 & \sigma_{F} & 0 & 0 & 0 & \sigma_{Par_{F}}^{2} & 0 & 0\\ 0 & 0 & 0 & \sigma_{S} & 0 & 0 & 0 & \sigma_{Par_{S}}^{2} & 0\\ 0 & 0 & 0 & 0 & \sigma_{T} & 0 & 0 & 0 & \sigma_{Par_{T}}^{2} \end{array}\right]$$

with σ_*M*_, for example, denoting the covariance between the actor and partner effect of the mother, and the following residual covariance matrix Θ:

$$\text{ψ =}\left[\begin{array}{cccccccccccc} \sigma_{MF}^{2} & 0 & 0 & \sigma_{M/F} & 0 & 0 & 0 & 0 & 0 & \sigma_{M/T} & 0 & 0\\ 0 & \sigma_{MS}^{2} & 0 & 0 & 0 & 0 & \sigma_{M/S} & 0 & 0 & 0 & 0 & 0\\ 0 & 0 & \sigma_{MT}^{2} & 0 & 0 & 0 & 0 & 0 & 0 & 0 & 0 & 0\\ \sigma_{M/F} & 0 & 0 & \sigma_{FM}^{2} & 0 & 0 & 0 & 0 & 0 & 0 & 0 & 0\\ 0 & 0 & 0 & 0 & \sigma_{FS}^{2} & 0 & 0 & \sigma_{F/S} & 0 & 0 & 0 & 0\\ 0 & 0 & 0 & 0 & 0 & \sigma_{FT}^{2} & 0 & 0 & 0 & 0 & \sigma_{F/T} & 0\\ 0 & \sigma_{M/S} & 0 & 0 & 0 & 0 & \sigma_{SM}^{2} & 0 & 0 & 0 & 0 & 0\\ 0 & 0 & 0 & 0 & \sigma_{F/S} & 0 & 0 & \sigma_{SF}^{2} & 0 & 0 & 0 & 0\\ 0 & 0 & 0 & 0 & 0 & 0 & 0 & 0 & \sigma_{ST}^{2} & 0 & 0 & \sigma_{T/S}\\ 0 & 0 & \sigma_{M/T} & 0 & 0 & 0 & 0 & 0 & 0 & \sigma_{TM}^{2} & 0 & 0\\ 0 & 0 & 0 & 0 & 0 & \sigma_{F/T} & 0 & 0 & 0 & 0 & \sigma_{TF}^{2} & 0\\ 0 & 0 & 0 & 0 & 0 & 0 & 0 & 0 & \sigma_{T/S} & 0 & 0 & \sigma_{TS}^{2} \end{array}\right]$$

with σ_*M*/*F*_, for example, denoting the covariance between the M-F and F-M relationship effects.

By adding structured means to the model the means of the SRM-effects are being defined. In the four-person SRM model with one indicator per relationship, we have in total 21 SRM means (1 family mean, 4 actor means, 4 partner means, and 12 relationship means), while only 12 means of the 12 dyadic measurements are observed. Therefore, restrictions are typically applied to the means such that the mean actor effects sum up to zero, the mean partner effects sum up to zero, and the mean SRM relationship effects sum up to zero for a given actor or a given partner. The family mean is simply defined as the average of the 12 dyadic measurements. These constraints are needed for model identification. By doing so the SRM-effects are unambiguously defined, a prerequisite for estimating the factor scores.

Alternatively, a weighted sum score can be obtained for each SRM-effect using the raw data. These scores are obtained by organizing the raw dyadic values for each of the relation-specific measures from each family in a two-way table in which the rows are the actors and the columns the partners (see Table 1). This table can be seen as a two-way ANOVA design, therefore the obtained scores are called ANOVA scores in this paper. The family effect (*Fam*) or grand mean of each family is defined as the average of the 12 dyadic measurements. For the calculation of the actor and partner effects, it is important to point out that the table contains empty cells. These empty cells are present since the round-robin design that we consider does not include self-ratings. If self-ratings were to be allowed, the row mean could be considered as an estimate of the actor effect and the column mean as an estimate of the partner effect (Cook and Kenny, 2004). The ANOVA score of the actor effect and partner effect for role *i* is estimated by weighing the respective row and column mean, and the grand mean, using the number of persons in the family (*n*; equal to 4 in the setting that we consider) i.e.,

(3) $$\begin{array}{l} Act_{i}={\left(n-1\right)}^{2}/\left[n\left(n-2\right)\right]\text{row }{\text{mean}}_{i}\\ \;+\left(n-1\right)/\left[n\left(n-2\right)\right]\text{column }{\text{mean}}_{i}\\ \;-\left(n-1\right)/\left(n-2\right)\text{grand mean} \end{array}$$

(4) $$\begin{array}{l} Par_{i}={\left(n-1\right)}^{2}/\left[n\left(n-2\right)\right]\text{column }{\text{mean}}_{i}\\ \;+\left(n-1\right)/\left[n\left(n-2\right)\right]\text{row }{\text{mean}}_{i}\\ \;-\left(n-1\right)/\left(n-2\right)\text{grand mean} \end{array}$$

A detailed derivation of these formulas for the SRM effects can be found in the Appendix. Importantly, if one of the dyadic measurements is missing no ANOVA-score can be obtained.

**Table 1:** Raw dyadic scores according to a two-way ANOVA design.

	**Partner**				
**Actor**	**Mother**	**Father**	**Target**	**Sibling**	**Row mean**
Mother		*X*_*MF*_	*X*_*MT*_	*X*_*MS*_	*Row Mean Mother*
Father	*X*_*FM*_		*X*_*FT*_	*X*_*FS*_	*Row Mean Father*
Target	*X*_*TM*_	*X*_*TF*_		*X*_*TS*_	*Row Mean Target*
Sibling	*X*_*SM*_	*X*_*SF*_	*X*_*ST*_		*Row Mean Sibling*
	*Column Mean Mother*	*Column Mean Father*	*Column Mean Target*	*Column Mean Sibling*	*Grand Mean*

## 3. SRM-effects of support

### 3.1. RADAR-Y

The RADAR study has been approved by the Medical Ethical Committee of Utrecht University Medical Centre (Netherlands). Participants of the RADAR-Y study were four-member families of 497 adolescents, who completed questionnaires during six annual home visits. Before the start of the study, adolescents and their parents received written information about the study and they provided their written informed consent in accordance with the Declaration of Helsinki. Parents gave written informed consent for the participation of their children. The sample of target adolescents consists of 283 boys and 214 girls. At the first wave, these adolescents were, on average 13 years old (*SD* = 0.5), their siblings were, on average, 15 years old (*SD* = 3.1), their mothers were, on average, 44 years old (*SD* = 4.5), and their fathers were, on average, 47 years old (*SD* = 5.1). The majority of the target adolescents were Dutch. At the time of the first wave of data collection, most adolescents were living with both parents, and most of their families were classified as having medium to high socioeconomic status. 466 families have data at the first three waves that we will consider.

The Network of Relationships Inventory [NRI; (Furman and Buhrmester, 1985)] support scale was used to measure dyadic support in family relationships. The eight items of the NRI are asked to each family according to a round robin design, where every family member rated the perceived support from every other family member using the same set of items. All items are rated according to a five-point scale ranging from one (not at all) to five (a lot). To perform the SRM-analysis, we will use only one measurement for each dyadic rating, as in most reported SRM-analyses. Therefore, one parcel was created by taking the average of the eight items. Missing values in the so obtained 12 dyadic measurements of support are present in at least one dyadic measurement for 22% of the families.

The proactive/reactive aggression (PRA; self-report) was used to measure the target adolescent's perception of victimhood of physical aggression, victimhood of relational aggression, physical aggression and relational aggression. The measures of victimhood of physical (*M* = 4.91, *SD* = 2.83) and relational aggression (*M* = 8.80, *SD* = 4.55) are taken from wave one, the perceived support measurements from wave two, physical (*M* = 8.98, *SD* = 4.89) and relational aggression (*M* = 19.91, *SD* = 8.46) as reported by the target adolescent from the third wave.

### 3.2. SRM analysis of perceived support

First an SRM with structured means, shown in Figure 1, was fitted to the RADAR-Y support data obtained at the second wave using the *R*-package lavaan (Rosseel, 2012), hereby relying on FIML estimation. The model yields an excellent fit [$\chi_{\left(47\right)}^{2}=76.262$, *p* = 0.004, CFI = 0.983, SRMR = 0.050, RMSEA = 0.037]. The family variance, the actor variance for all roles, the partner variance of the father and the sibling, and all relation-specific variances (confounded with the residual error here) were all significant (based on a Wald test).

The family effect explained 7–19% of the total variance and the actor variance accounted for 19–56% of the total variance in the dyadic measurements of support. The explained variance in dyadic support explained by the partner effects ranged from 2 to 21%. We can thus conclude that perceived support depends considerably on the characteristics of the actor (i.e., the perceiver). But what are possible antecedents and consequences of these SRM-effects?

### 3.3. A naive ANOVA-based analysis of the support data

**Figure 2:**
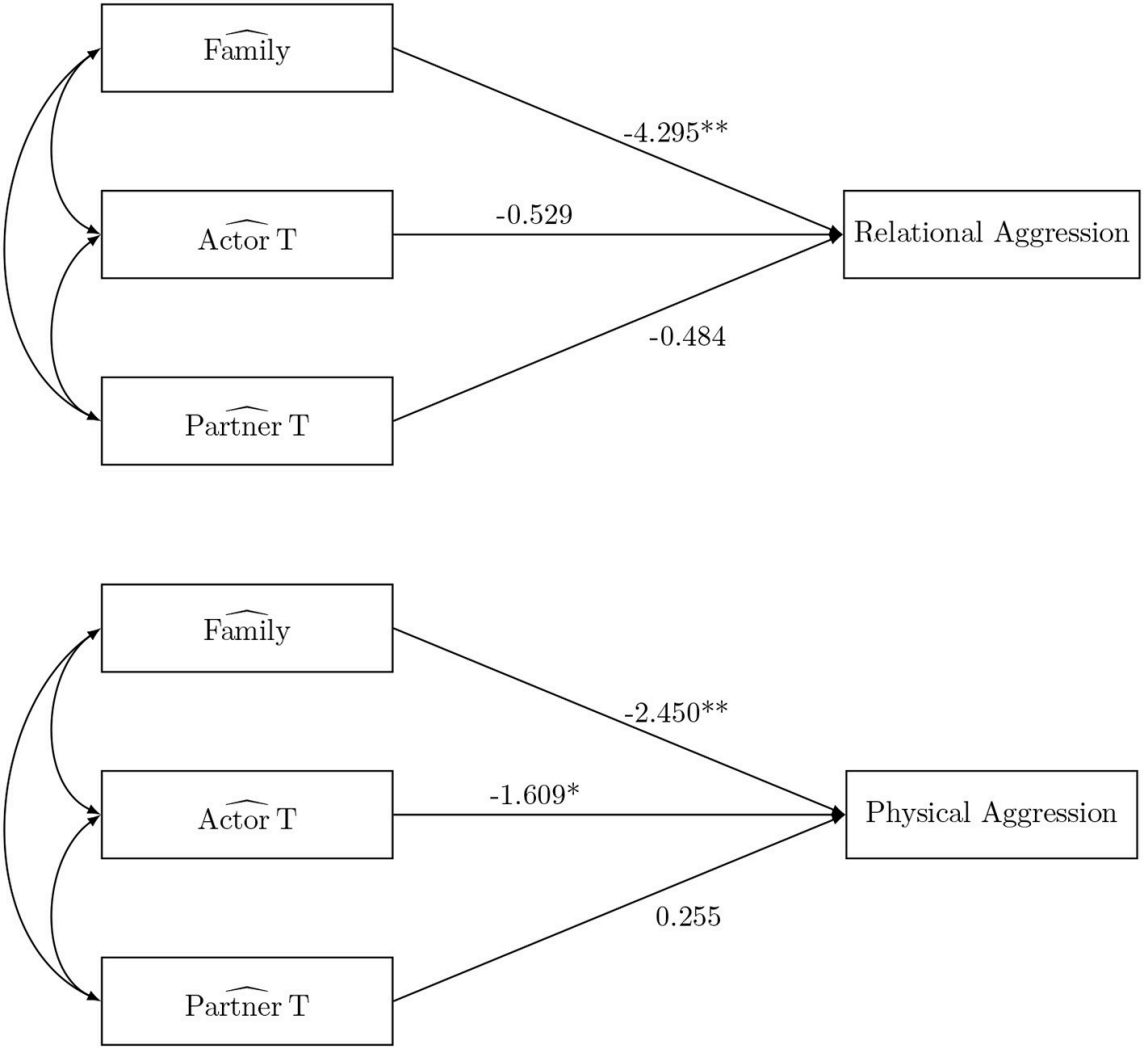
The social relations model components as predictor in the naive ANOVA analysis. ^*^p < 0.05, ^**^p < 0.01, and ^***^p < 0.001.

The ANOVA-based approach is used to estimate the SRM-effects in each family. Table 2 shows the sample mean and sample variance of the 9 ANOVA-based SRM-effects of interest. Note that there were 362 families with complete dyadic measurements of support at wave 2. The ANOVA-scores are subsequently used to estimate relationships with external variables. First, we can investigate the consequences of the SRM-effects of support (Figure 2). Physical and relational aggression reported by the target adolescent at wave 3 are regressed on the family effect and the target adolescent's actor and partner effect. The estimated regression coefficients can be found in Figure 2. Significant negative effects of the family effect of perceived support (i.e., overall shared supportive climate) at wave 2 on the two adverse outcomes are observed. That is, the larger the perceived support in the family, the smaller the amount of self-reported physical and relational aggression. Furthermore, the target adolescent's perceived support from other family members at wave 2 has a negative effect on later physical aggression. That is, the more the target adolescent perceives support, the smaller the amount of physical aggression he/she will show. Note that we can merely talk about associations, here and in the remainder of the paper, and not about causal effects.

**Table 2:** Means, variances, and number of observations of the ANOVA scores.

**SRM-effect**	**Mean**	**Variance**	***n***
Family effect	3.465	0.104	362
Actor T	0.003	0.148	362
Partner T	−0.200	0.058	362
Actor M	0.143	0.116	362
Partner M	0.283	0.059	362
Actor F	−0.101	0.124	362
Partner F	0.027	0.093	362
Actor S	−0.044	0.144	362
Partner S	−0.110	0.061	362

**Figure 3:**
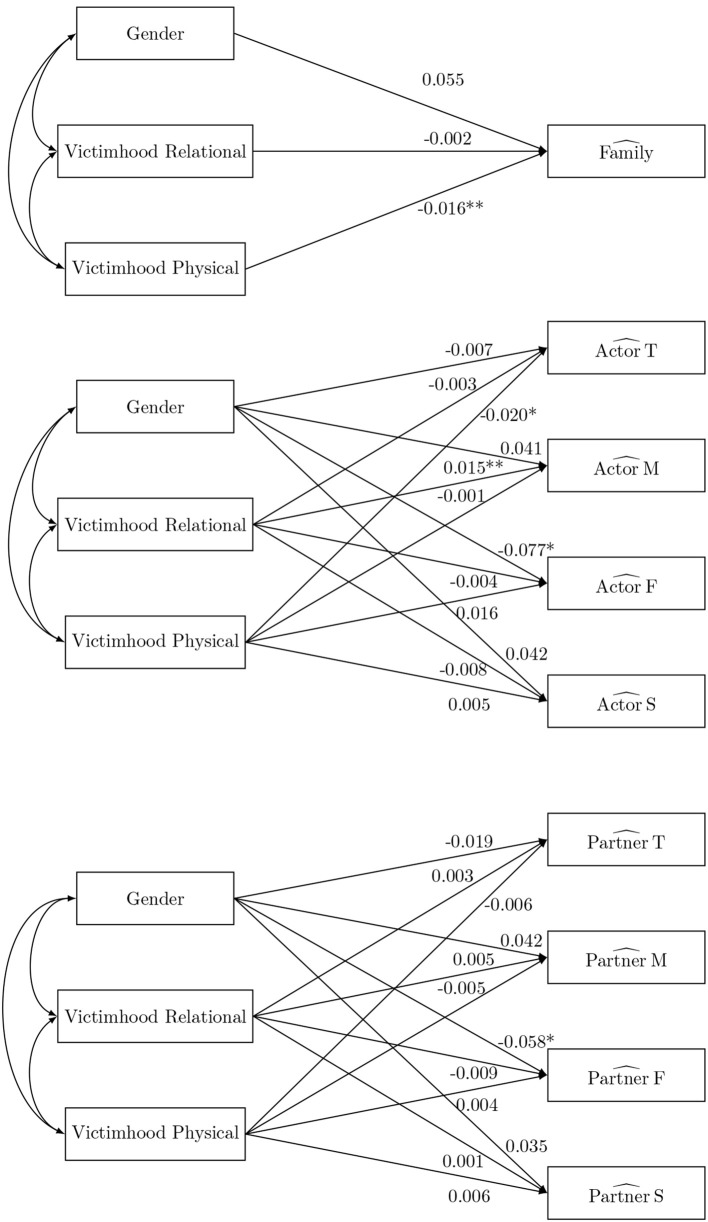
The social relations model components as outcome in the naive ANOVA analysis. ^*^p < 0.05, ^**^p < 0.01, and ^***^p < 0.001).

Second, possible antecedents (the target adolescent's gender, victimhood of relational and physical aggression at wave 1) of the SRM-effects of perceived support at wave 2 are investigated (Figure 3). The first model considers the family effect as dependent variable, the second considers all the actor effects simultaneously and the third considers all the partner effects simultaneously. This separation of the family effect from the actor effects (partner effects, respectively) is needed due to the aforementioned linear dependency among the SRM-effects. Furthermore, it may seem counterintuitive to study the effect of the target adolescent's gender on all the actor effects (partner effects, respectively). However, it is a necessity since the regression coefficients must adhere to the constraint that conditional on the predictor the mean actor effects (partner effects, respectively) sum to zero for the aforementioned identification reasons. The estimated path coefficients can be found in Figure 3. There is a significant effect of self-reported victimhood of relational aggression on the family effect of support, which indicates that higher levels of victimization are related to overall lower familial support. Also a positive effect of victimhood of relational aggression is observed on the actor effect of the mother, showing that the mother tends to perceive more support when the target adolescent reports higher levels of victimization. There is also a negative effect of victimhood of physical aggression on the actor effect of the target adolescent, indicating that the adolescent perceives less support when they report higher levels of victimization. There is no impact of gender on the family effect of support nor on any of the actor effects. However, there is an impact of gender on both the actor and partner effect of the father. That is, in families where the target adolescent is a girl the father will experience less support than in families where the target adolescent is a boy. Additionally, he will also be perceived as less supportive in families where the target adolescent is a girl than in families where the target adolescent is a boy.

The ANOVA-based approach presented here is often used but limited. First, it entails a limited information analysis instead of a full information analysis, that is only families with complete data are included. In our data, SRM-effects are missing in 22% of the families. Second, the unbiasedness of the estimator of the path coefficients is questionable (Croon and van Veldhoven, 2007; Lüdtke et al., 2018). Therefore, we introduce FSR approaches as an alternative in the next section.

## 4. Factor score estimation methods

There are multiple methods for the estimation of factor scores. In this section we will briefly review the two most commonly used approaches: the regression and Bartlett. We also discuss the full information maximum likelihood (FIML) based extension of the regression factor score (Estabrook and Neale, 2013) and the Bartlett factor score.

Consider the following multi-factor model, with a vector of continuous indicators, **x**, and a vector of latent variables, **η**,

(5) $$\begin{array}{r} \text{x}=\alpha +\Lambda \eta +\epsilon \end{array}$$

where the measurement error **ϵ** is multivariate normal distributed with covariance matrix Θ. The factors are multivariate normal with covariance matrix Ψ, and factor loading matrix is Λ. Consequently, the covariance structure of this model, assuming independence between the factors and the residuals, can be estimated as

(6) $$\begin{array}{r} \hat{\Sigma }=\hat{\Lambda }\hat{\text{Ψ}}{\hat{\Lambda }}^{\prime }+\hat{\Theta } \end{array}$$

The estimation of the parameters of the measurement part is the first step for estimating the factor scores, which are the predicted values of **η** for each observation.

### 4.1. Regression factor score

The most ubiquitous method applied is the Thurstone-Thomson or regression factor score estimator (Thomson, 1934; Thurstone, 1935). This method is also known as the empirical Bayes predictor (Gill and Swartz, 2001; Skrondal and Rabe-Hesketh, 2004). In general, the regression method defines the factor score coefficient (FSC) as the product of three terms: the estimated covariance matrix of the factors ($\hat{\text{Ψ}}$), factor loading matrix ($\hat{\Lambda }$) and model-implied covariance matrix ($\hat{\Sigma }$). This weight matrix is then multiplied with the difference between the observed indicators (***x***) and their estimated means ($\hat{\mu }$).

(7) $$\begin{array}{r} \hat{\eta }=\hat{\text{Ψ}}{\hat{\Lambda }}^{\prime }{\hat{\Sigma }}^{-1}\left(x-\hat{\mu }\right) \end{array}$$

Alternatively, the FSC of the regression factor score estimator (*FSC*_*R*_) can be specified as

(8) $$\begin{array}{r} FSC_{R}={\left({\hat{\text{Ψ}}}^{-1}+{\hat{\Lambda }}^{\prime }{\hat{\Theta }}^{-1}\hat{\Lambda }\right)}^{-1}{\hat{\Lambda }}^{\prime }{\hat{\Theta }}^{-1} \end{array}$$

This estimator is conditionally biased (Bentler and Yuan, 1997; Skrondal and Rabe-Hesketh, 2004) and only results in unbiased regression coefficients when it is used as an independent variable (Skrondal and Laake, 2001; Devlieger et al., 2016). Devlieger et al. (2016) have shown that when the regression factor scores are used as independent variable for *y*, the estimated regression coefficients $\hat{\beta }$ are related to the true values **γ** as follows:

(9) $$\begin{array}{l} \begin{array}{l} E\left[\hat{\beta }\right]=\frac{cov\left(\hat{\eta },y\right)}{var\left(\hat{\eta }\right)}\\ ={\left(FSC_{R}\hat{\Sigma }FSC_{R}^{\prime }\right)}^{-1}FSC_{R}\hat{\Lambda }\hat{\text{Ψ}}\gamma \\ ={\left(FSC_{R}\hat{\Lambda }\hat{\text{Ψ}}\right)}^{-1}FSC_{R}\hat{\Lambda \hat{\text{Ψ}}}\gamma \\ =\gamma \end{array} \end{array}$$

Alternatively, the maximum likelihood framework can be considered. Here, the factor scores are obtained by optimizing the likelihood of the factor score estimates given the observed data (Skrondal and Rabe-Hesketh, 2004; Estabrook and Neale, 2013):

(10) $$\begin{array}{r} L\left(\hat{\eta }|x\right)=L\left(\hat{\eta }\right)L\left(x|\hat{\eta }\right) \end{array}$$

Since it makes use of all available data, the likelihood approach makes it possible to estimate factor scores in the presence of missing values. Indeed, in the presence of missingness the FIML estimates are simply obtained by adjusting the *FSC*_*R*_ so that residual covariance matrix $\hat{\Theta }$ and the factor loading matrix $\hat{\Lambda }$ only contain the rows and columns of the non-missing (n-m) indicators x, i.e.,

(11) $$\begin{array}{r} \hat{\eta }={\left({\hat{\text{Ψ}}}^{-1}+{\hat{\Lambda }}_{n-m}^{\prime }{\hat{\Theta }}_{n-m}^{-1}{\hat{\Lambda }}_{n-m}\right)}^{-1}{\hat{\Lambda }}_{n-m}^{\prime }{\hat{\Theta }}_{n-m}^{-1}\left({\text{x}}_{n-m}-\hat{\mu_{n-m}}\right) \end{array}$$

We refer to this estimator as the regression Full Information Maximum Likelihood (FIML) estimator.

### 4.2. Bartlett factor score

Conditional unbiasedness is an appropriate criterion when the factor scores are used as dependent variables. Such conditional unbiased estimator was introduced by Bartlett (1937), the Bartlett factor scores. Here, the FSC is obtained using the estimated factor loading matrix ($\hat{\Lambda }$) and residual covariance matrix ($\hat{\Theta }$),

(12) $$\begin{array}{r} \hat{\eta }={\left({\hat{\Lambda }}^{\prime }{\hat{\Theta }}^{-1}\hat{\Lambda }\right)}^{-1}{\hat{\Lambda }}^{\prime }{\hat{\Theta }}^{-1}\left(x-\hat{\mu }\right) \end{array}$$

Typically, $\hat{\Theta }$ is assumed to be diagonal and positive definite, but in practice this is often not the case. Therefore, Bentler and Yuan (1997) considered an alternative for the Bartlett estimator, by replacing the residual covariance matrix ($\hat{\Theta }$) with the model implied covariance matrix ($\hat{\Sigma }$). This generalized least squares estimator results in factor score estimates equivalent to those of the Bartlett estimator.

(13) $$\begin{array}{r} \hat{\eta }={\left({\hat{\Lambda }}^{\prime }{\hat{\Sigma }}^{-1}\hat{\Lambda }\right)}^{-1}{\hat{\Lambda }}^{\prime }{\hat{\Sigma }}^{-1}\left(x-\hat{\mu }\right) \end{array}$$

Another issue arises when the factor loading matrix is not full rank. Then the product ${\hat{\Lambda }}^{\prime }{\hat{\Theta }}^{-1}\hat{\Lambda }$ will be singular and non-invertible. In that case the Moore-penrose generalized pseudo-inverse can offer a solution (Bentler and Yuan, 1997; Neudecker and Satorra, 2003).

(14) $$\begin{array}{r} \hat{\eta }={\left({\hat{\Lambda }}^{\prime }{\hat{\Theta }}^{-1}\hat{\Lambda }\right)}^{+}{\hat{\Lambda }}^{\prime }{\hat{\Theta }}^{-1}\left(x-\hat{\mu }\right) \end{array}$$

where the superscript + denotes the generalized pseudo-inverse. As mentioned before this particular issue also arises with the SRM.

The Bartlett estimator yields unbiased regression coefficients when it is applied as a dependent variable (Skrondal and Laake, 2001; Devlieger et al., 2016). Devlieger et al. (2016) have shown that when the Bartlett factor scores are used as dependent variable of *z*, the estimated regression coefficients $\hat{\beta }$ are related to the true values **γ** as follows:

(15) $$\begin{array}{r} \begin{array}{l} E\left[\hat{\beta }\right]=\frac{cov\left(z,\hat{\eta }\right)}{var\left(z\right)}\\ ={\left({\hat{\Lambda }}^{\prime }{\hat{\Theta }}^{-1}\hat{\Lambda }\right)}^{-1}{\hat{\Lambda }}^{\prime }{\hat{\Theta }}^{-1}\hat{\Lambda }\gamma \\ =FSC_{B}\hat{\Lambda }\gamma \end{array} \end{array}$$

To obtain unbiased regression coefficients the product of the FSC of the Bartlett factor score estimator (*FSC*_*B*_) and the factor loading matrix should equal the identity matrix. Since the factor loading matrix of the SRM model is not full rank, we are forced to use the generalized inverse as in (14) for *FSC*_*B*_. As we will see later, the product of the *FSC*_*B*_ of the SRM-effects and its factor loading matrix will not necessarily result in an identity matrix.

Also for the Bartlett estimator, the maximum likelihood framework can be considered. As pointed out by Skrondal and Rabe-Hesketh (2004) the Bartlett factor score estimates can be obtained by optimizing the likelihood of the observed data given the factors

(16) $$\begin{array}{r} L\left(x|\hat{\eta }\right) \end{array}$$

To obtain the Bartlett FIML estimates in the presence of missing data the *FSC*_*B*_ can easily be adjusted so that residual covariance matrix $\hat{\Theta }$ and the factor loading matrix $\hat{\Lambda }$ only contain the rows and columns of the non-missing (n-m) indicators x, i.e.,

(17) $$\begin{array}{r} \hat{\eta }={\left({\hat{\Lambda }}_{n-m}^{\prime }{\hat{\Theta }}_{n-m}^{-1}{\hat{\Lambda }}_{n-m}\right)}^{-1}{\hat{\Lambda }}_{n-m}^{\prime }{\hat{\Theta }}_{n-m}^{-1}\left({\text{x}}_{n-m}-{\hat{\mu }}_{n-m}\right) \end{array}$$

### 4.3. Performance of regression and bartlett with the SRM

The unbiasedness of the estimated regression coefficients when the regression factor score (6) is used as predictor, or the Bartlett factor score (12) is used as outcome, has been shown analytically and in simulation studies in simple settings (Devlieger et al., 2016). The standard errors of the regression coefficients of the subsequent path or regression analyses are however underestimated by FSR, since it is implicitly assumed that the factor scores are measured without error. An exception occurs when there is no relation present between the factor scores and the (in)dependent variable. In that case, no standard error bias is present in the FSR methods (Devlieger et al., 2016).

In the next section, we run a simulation-study to assess the performance of regression and Bartlett factor scores in the SRM context. First, the use of a generalized inverse for the Bartlett factor scores needs further exploration. Does it lead to unbiased regression coefficients? Second, while the performance of SEM FIML has been studied before (Cham et al., 2017) the performance of the FSR FIML-based approaches in the presence of missing data have never been investigated to our knowledge. Under what assumptions do we obtain valid inference with FSR under missingness? Two missing data scenario's will be distinguished. In the first scenario, missingness in the dyadic measurements depends on the other observed dyadic measurements. The missing values in both SEM and FSR can be considered MAR in that scenario, since the variables responsible for the missing values are taken into account. In the second scenario, missingness in the dyadic measurements depends on the observed outcome or predictor that is associated with the SRM-effects. Then, only under SEM the missing values can be considered MAR. Under FSR, these are missing not at random (MNAR), since the variables explaining the missingness are not taken into account during the estimation.

Based on the theoretical background and previous simulation studies (Devlieger et al., 2016), we hypothesize that when the SRM-effects are used as independent variable:

1. In the complete-case setting SEM and regression FSR result in unbiased regression coefficients for the effect of SRM-components on an outcome;
2. In the presence of missing dyadic measurements:
  - SEM FIML and regression FIML result in unbiased regression coefficients if missingness in the dyadic measurements only depends on other observed dyadic measurements;
  - Only SEM FIML results in unbiased regression coefficients if missingness also depends on the observed outcome;
3. Bartlett (FIML) and ANOVA result in biased estimates in both the complete case-setting and in the presence of missingness;
4. When the underlying true regression coefficient is 0, the type I error rate of all the methods is correct;
5. SEM shows the best coverage.

When the SRM-effects are used as dependent variable, we hypothesize:

1. In the complete-case setting SEM results in unbiased regression coefficients for the effect of a predictor on SRM-components;
2. In the presence of missing dyadic measurements:
  - SEM FIML result in unbiased regression coefficients if missingness depends on other observed dyadic measurements;
  - SEM FIML results in unbiased regression coefficients if missingness also depends on the observed predictor;
3. Regression (FIML), Bartlett and ANOVA result in biased estimates in both the complete case-setting and in the presence of missingness;
4. When the underlying true regression coefficient is 0, the type I error rate of all the methods is correct;
5. SEM shows the best coverage.

## 5. Simulation

In the simulation study we compare the performance of regression (FIML) and Bartlett (FIML) with SEM and the ANOVA scores. All analyses were performed using the statistical software *R* (R Core Team, 2017). The complete-case regression and Bartlett factor scores are available in the *R*-package lavaan (Rosseel, 2012), while only the regression FIML factor scores are available in Mplus (Muthén and Muthén, 2012). Since neither package contains the Bartlett FIML expression presented in this paper, we programmed the calculation of both the regression FIML and Bartlett FIML in *R*. The steps described in this section can easily be replicated by the reader using the Supplementary Material. Additional information on the performance of the methods can also be found in the Supplementary Material. Note that in the body of text we do not provide tables with results for every single SRM-effect. We rather opted to present summary tables with stars indicating poor, moderate or good performance over all effects, this to give a more global impression of the performance of the different methods. The performance of the different methods is compared in terms of bias, coverage and precision. First, the bias was assessed using a z-score, comparing the underlying true value with the median of the estimated regression coefficients across simulations. Note that we used the median instead of the mean to minimize the impact of outliers. The empirical coverage was calculated as the proportion of times the 95% confidence interval of the estimated path coefficient on basis of its estimated standard error did contain the true underlying value. Further, the precision was calculated using the median absolute deviation (MAD) from the true value, which is a more robust measure than the mean squared error (MSE). The performance of a method is seen as poor, when the method shows bias, under-or overcoverage and imprecision for multiple parameters. The performance is seen as moderate when the method only shows bias, under-or overcoverage and imprecision for only a few parameters. The performance is seen as good when the method performs excellent for all the parameters. We consider two conditions: first the SRM-components as antecedents and second as consequences.

### 5.1. SRM as predictor

#### 5.1.1. Technical details

**Figure 4:**
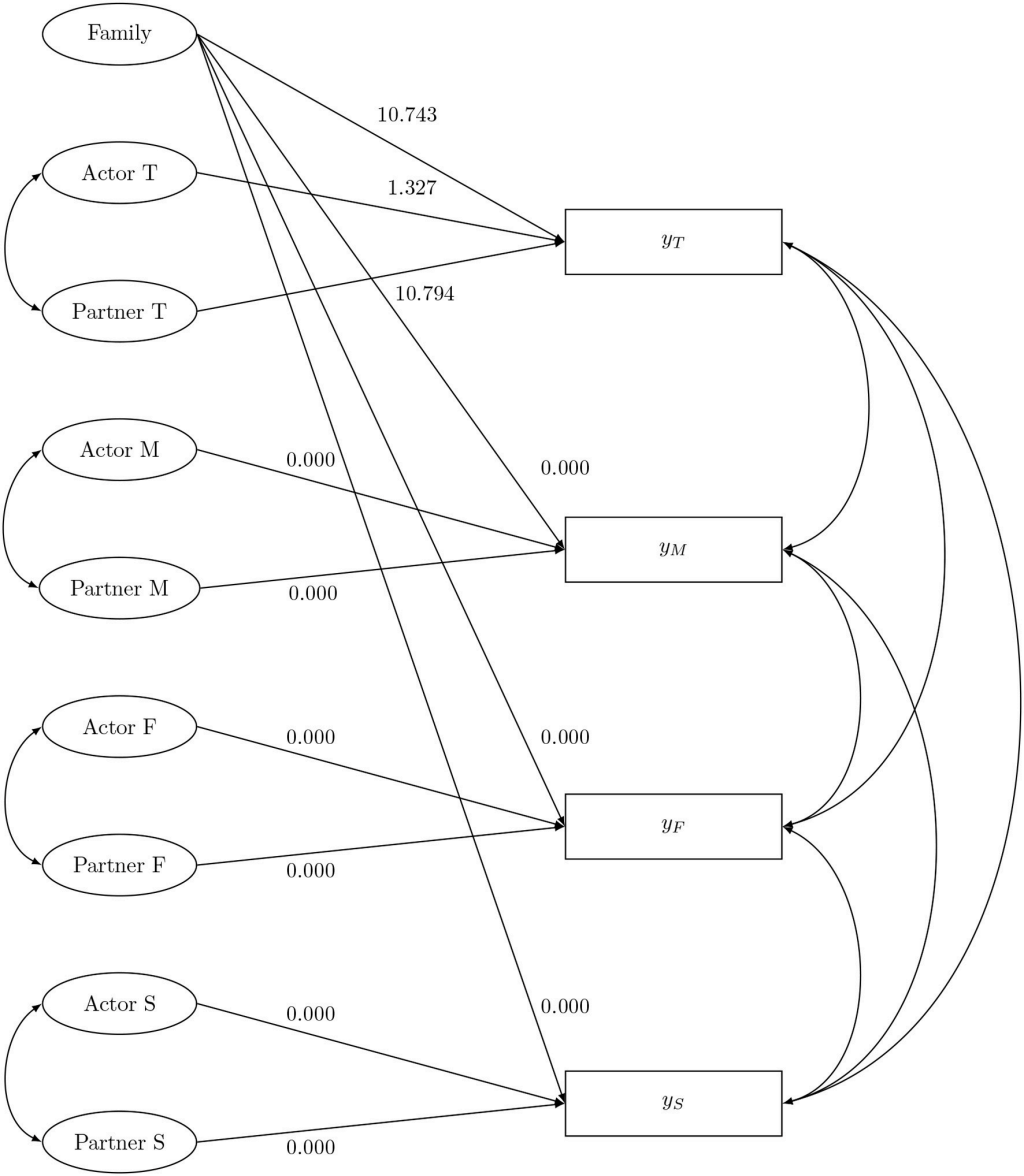
The social relations model components as predictor: data-generating model.

In this condition the SRM-components of four-member families are used as independent variables. We assume a SEM model with four regressions as depicted in Figure 4. Each outcome variable *y*_*i*_ is role-specific. Therefore, only the following SRM-effects are considered as predictors of *y*_*i*_: the family effect, the actor effect of role *i* and the partner effect of role *i*. In this data-generating model the variance of the family effect is set to 1, the variances of all the perceiver effects are set to 1, the variances of all the partner variances are set to 0.5 and the variances of all the relationship-effects are set to 1.5. Additionally, the generalized reciprocities are set to 0.05 or −0.05 and the dyadic reciprocities are all set tot 0.02. The data were randomly generated from the model-implied multivariate normal distribution using the *R*-package lavaan. A complete-case scenario and two missing data scenarios are considered. In the first missing data scenario, missingness in some of the dyadic measurements is related to other dyadic measurements. In detail, missing values in the dyadic measurements *X*_*TM*_ and *X*_*TF*_ were randomly generated with a missing rate of 25% when *X*_*TS*_ had a value that was smaller than its first quartile. In the second scenario missingness is related to the outcome. Missing values were then randomly generated in *X*_*TM*_, *X*_*TF*_, and *X*_*TS*_ with a missing rate of 25%, when *y*_*T*_ had a value that was smaller then its first quartile. For each scenario 1,000 simulations were performed. We considered sample sizes of either *n* = 50 or *n* = 500 four-person families.

#### 5.1.2. Results

An overview of the performance of the methods in the complete-case scenario is presented in the upper part of Table 3. The SEM and regression factor scores result in unbiased regression coefficients for both sample sizes, but the Bartlett and the ANOVA scores generally do not. Note that the latter two methods do however result in unbiased estimators for zero-effects. None of the methods displays a good coverage probability in small samples. In large samples SEM shows the aspired coverage probability for all the effects, while the FSR methods only display good coverages for the zero-effects. For the non-zero effects, the coverage of the FSR methods can be enhanced by estimating the standard errors using bootstrap (results not shown). More specifically, sampling with replacement from the families is used. In the latter, it is acknowledged that the factor scores are estimated with error, rather than being treated as fixed. Note that the confidence intervals in SEM can also be estimated using bootstrap or profile-likelihood confidence intervals, which would enhance its coverage probability in small samples as well (Pek and Wu, 2015). The regression methods have a better precision than SEM in small samples, but with increasing sample size this discrepancy becomes negligible. In the settings with small samples SEM did not converge properly in 7.40% of the simulations when simultaneously modeling the SRM and its consequences. Non-convergency issues arose when the fitted covariance matrix was not positive definite, which might indicate improper results such as negative variances. No convergence issues were observed for the FSR methods that only require to fit the SRM in the first step. Non-convergency solutions were excluded from all further analyses.

**Table 3:** Comparison of the methods: SRM as predictor.

	***n*** = **50**			***n*** = **500**		
**Complete-case**	**Bias**	**Coverage**	**Precision**	**Bias**	**Coverage**	**Precision**
SEM	***	**	*	***	***	***
Regression	**	**	**	***	**	***
Bartlett	*	*	***	*	*	***
ANOVA	*	*	***	*	*	***
**MISSINGNESS RELATED TO THE PREDICTOR**						
SEM FIML	***	**	*	***	***	**
Regression FIML	**	**	*	***	**	**
Bartlett FIML	*	**	**	*	**	**
ANOVA	*	**	**	*	*	*
**MISSINGNESS RELATED TO THE OUTCOME**						
SEM FIML	***	**	**	***	***	**
Regression FIML	**	**	**	**	**	**
Bartlett FIML	*	**	**	*	**	***
ANOVA	*	**	**	*	*	**

* *poor performance*,

** moderate performance, and

*** *good performance*.

The middle part of Table 3 presents the performance of the methods in the presence of missingness related to other dyadic measurements. An average missing rate of about 14% is observed. Again, unbiased regression coefficients are found using SEM FIML and regression FIML. SEM FIML shows the best coverage probabilities in both small and large samples. Again, by estimating the standard errors of the path coefficients with a bootstrap, the coverage of regression FIML could be enhanced. The performance of the SEM and FSR is also comparable in terms of precision in small and large samples. In the small samples SEM FIML did not converge properly in 8.10% of the simulations.

An overview of the methods, in the presence of missing values related to the outcome can be found in the lower part of Table 3. An average missing rate of about 14% is observed. Unbiased regression coefficients are found with SEM FIML and regression FIML in the small samples. In the large samples only SEM FIML results in unbiased regression coefficients, while regression FIML results in some slight bias. SEM FIML shows the best coverage probabilities in both small and large samples. The performance of SEM FIML and regression FIML is also comparable in terms of precision in small and large samples. Again, 7.10% of SEM FIML did not converge properly in the small samples.

In sum, all our hypotheses are confirmed. We conclude that there is no method that outperforms SEM (FIML) when the SRM-components are used as predictor. But if SEM fails to converge regression FIML is an acceptable alternative (provided that missingness in the dyadic scores only depend on other measured dyadic scores), since no convergency issues were encountered when fitting the SRM that was used as base model for the computation of the factor scores. Alternatively, one could use the estimates from regression FIML as starting values in SEM FIML to overcome the convergency issues.

### 5.2. SRM as outcome

#### 5.2.1. Technical details

**Figure 5:**
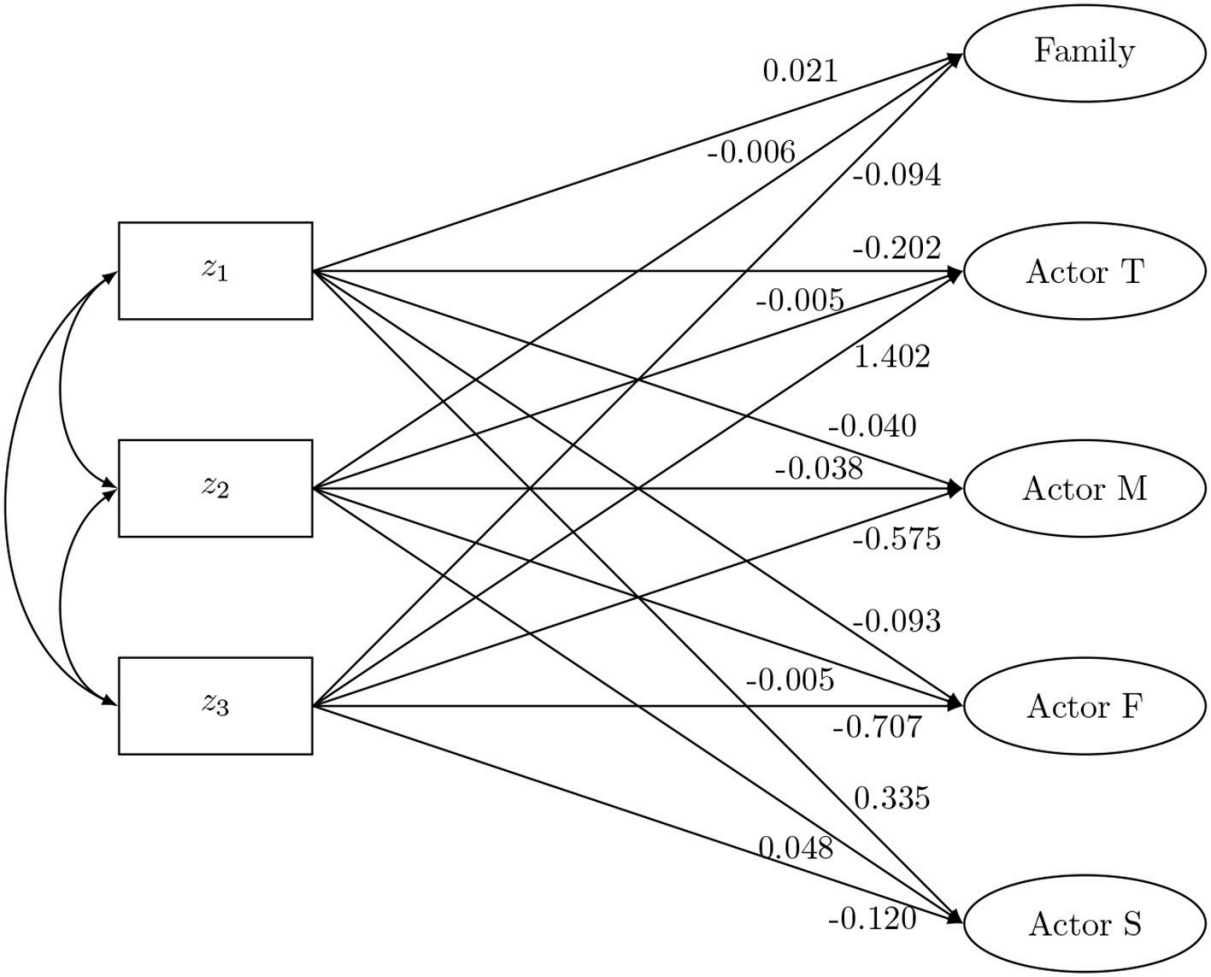
The social relations model components as outcome: data-generating model.

In this second condition, the SRM-components are used as dependent variables. Data were generated from the SEM model shown in Figure 5. Here, the SRM-components are regressed on three predictors: *z*_1_, *z*_2_, and *z*_3_. Zero-effects are assumed between the predictors and the partner effects. This SEM can be viewed as a multiple indicators multiple causes (MIMIC) model, and is equivalent to a multiple group CFA with equal latent variances, but different latent means across groups (Kim and Cao, 2015). Note that the regression coefficients were chosen in such a way that conditional on the predictor the sum of the actor effects (partner effects, respectively) equals zero. The data-generating model contains the same values for the variances and reciprocities as in the previous section. In this condition a complete-case scenario and two missingness scenarios are considered as well. In the first missingness scenario, missingness is related to other dyadic measurements. Here, missing values were randomly generated in the dyadic measurements *X*_*TM*_, *X*_*TF*_, *X*_*TS*_, *X*_*FM*_, *X*_*FT*_, and *X*_*FS*_ with a missing rate of 40%, when *X*_*SM*_ had a value that was smaller then its first quartile. In the second scenario, missingness is related to the predictor. Missing values were randomly generated in *X*_*TM*_, *X*_*TF*_, *X*_*TS*_, *X*_*FM*_, *X*_*FT*_, and *X*_*FS*_ with a missing rate of 40%, when *z*_1_ had a value that was smaller then its first quartile. Missing values were also generated in *X*_*TM*_, *X*_*TF*_, *X*_*TS*_, *X*_*MF*_, *X*_*MT*_, and *X*_*MS*_, with a missing rate of 40%, when *z*_2_ had a value smaller then its first quartile. Note, simulations with a smaller missing rate (i.e., 25%) result in the same conclusions (results not shown). Again, 1,000 simulations were performed for each scenario. Sample sizes of either *n* = 50 or *n* = 500 four-person families were considered.

#### 5.2.2. Results

In the upper part of Table 4 an overview is presented of the performance in the complete-case scenario. SEM and the ANOVA scores result in unbiased regression coefficients for both sample sizes. The Bartlett and regression scores generally result in biased coefficients. None of the methods displays a good coverage probability in the small samples, however the under-coverage of SEM disappears in large samples. Both ANOVA and SEM have a comparable precision irrespective of the sample size.

**Table 4:** Comparison of the methods: SRM as outcome.

	***n*** = **50**			***n*** = **500**		
**Complete-case**	**Bias**	**Coverage**	**Precision**	**Bias**	**Coverage**	**Precision**
SEM	***	**	**	***	***	**
Regression	*	*	***	*	*	***
Bartlett	***	*	**	**	**	**
ANOVA	***	*	**	***	**	**
**MISSINGNESS RELATED TO THE OUTCOME**						
SEM FIML	***	***	**	***	***	**
Regression FIML	*	**	**	*	*	**
Bartlett FIML	**	**	**	*	**	**
ANOVA	*	**	*	*	**	*
**MISSINGNESS RELATED TO THE PREDICTOR**						
SEM FIML	***	***	**	***	***	**
Regression FIML	*	**	***	*	**	**
Bartlett FIML	**	**	***	*	**	***
ANOVA	***	**	*	***	***	*

* *poor performance*,

** moderate performance, and

*** *good performance*.

The middle part of Table 4 presents the results in the presence of missingness related to other dyadic measurements. An average missing rate of about 24% is observed. All methods show some bias in the small and large samples, except for SEM FIML. Furthermore, SEM FIML outperforms the other methods in coverage and precision as well.

In the lower part of Table 4 the results in the presence of missingness related to the predictor can be found. An average missing rate of about 45% is observed. SEM FIML and the ANOVA scores are the only methods that result in unbiased estimates. SEM FIML outperforms the ANOVA-based approach and Bartlett FSR in coverage and precision.

In sum, SEM (FIML) clearly outperforms all the other methods in this condition. However, it is important to stress that in the SEM-approach one needs to constrain the regression coefficients such that conditional on the predictor the actor effects (partner effects, respectively) sum to zero. Furthermore, almost all of our hypotheses are met, concluding that in this condition there is no suitable alternative for SEM. As suggested before, the observed bias in the Bartlett FSR may be explained by the relation between the estimated regression coefficients ($\hat{\beta }$) and the true value (γ), and the use of the generalized inverse in the factor score matrix. Indeed, when the factor loading and model-implied residual matrix from the simulation model are used, the following matrix is obtained:

(18) $${\left(\Lambda^{\prime }\Theta^{-1}\Lambda \right)}^{+}\Lambda^{\prime }\Theta^{-1}\Lambda =\left(\begin{array}{lllllllll} 0.667 & 0.167 & 0.167 & 0.167 & 0.167 & 0.167 & 0.167 & 0.167 & 0.167\\ 0.167 & 0.792 & -0.208 & -0.208 & -0.208 & 0.042 & 0.042 & 0.042 & 0.042\\ 0.167 & -0.208 & 0.792 & -0.208 & -0.208 & 0.042 & 0.042 & 0.042 & 0.042\\ 0.167 & -0.208 & -0.208 & 0.792 & -0.208 & 0.042 & 0.042 & 0.042 & 0.042\\ 0.167 & -0.208 & -0.208 & -0.208 & 0.792 & 0.042 & 0.042 & 0.042 & 0.042\\ 0.167 & 0.041 & 0.042 & 0.042 & 0.042 & 0.792 & -0.208 & -0.208 & -0.208\\ 0.167 & 0.041 & 0.042 & 0.042 & 0.042 & -0.208 & 0.792 & -0.208 & -0.208\\ 0.167 & 0.041 & 0.042 & 0.042 & 0.042 & -0.208 & -0.208 & 0.792 & -0.208\\ 0.167 & 0.041 & 0.042 & 0.042 & 0.042 & -0.208 & -0.208 & -0.208 & 0.792 \end{array}\right)$$

Since this product does not result in an identity matrix, bias will be present in the regression coefficients of the Bartlett FSR. As an example, Table 5 shows the expected biased values of the regression coefficients of the family effect due to the generalized inverse expression (18) and the mean of the estimated regression coefficients of the Bartlett approach in the complete-case scenario with sample size of 500. These values correspond closely.

A possible solution to avoid a generalized inverse would be to redefine the factor loading matrix. One could opt not to fix all loadings of the SRM-effects to one, but to redefine the factor loading matrix by restricting the average of the factor loadings for each effect to be one, i.e., effect-coding (Little et al., 2006). We do not recommend this approach, since we often encountered convergency issues and obtained more imprecise coefficients when using free factor loadings (results not shown). In conclusion, the results indicate that one should be careful when employing a generalized inverse in the calculation of the Bartlett factor scores, and SEM is the only reliable option when SRM-effects are used as outcome. Note that the ANOVA scores are a valuable alternative for SEM when no missingness is present.

**Table 5:** SRM as outcome, biased values of the regression coefficients of the family effect due to generalized inverse (G.I).

**Family effect**	**True γ**	**β with G.I**.	**Mean bartlett estimate**
*x*_1_	0.021	0.014	0.016
*x*_2_	−0.005	−0.004	−0.004
*x*_3_	−0.094	−0.063	−0.060

## 6. Case study

In this section the best performing methods according to the simulation study are illustrated using the aforementioned data from the RADAR-Y study.

### 6.1. SRM-components of perceived support as predictor

We first consider SRM-components as predictor, which allows us to investigate the consequences of the SRM-effects (see Figure 2). The simulation study indicated that both SEM FIML and regression FIML yield unbiased coefficients in this setting. We fitted a SEM model, with physical and relational aggression reported by the target adolescent at wave 3 regressed on the family effect, actor and partner effect of the target adolescent derived from the SRM that was fitted simultaneously. The fit was acceptable [$\chi_{\left(67\right)}^{2}=98.695$, *p* = 0.007, CFI = 0.984, SRMR = 0.048 RMSEA = 0.032]. The SRM fitted separately and discussed above, forms the basis for the estimation of the regression FIML factor scores. A summary of the regression coefficients obtained using SEM FIML and regression FIML can be found in Table 6. The results from the SEM FIML model and the regression FIML FSR are indeed similar.

**Table 6:** Casestudy: SRM-components as predictor.

	**Parameters**	**SEM FIML (s.e.)**	**Regression FIML (s.e.)**
**Relational**			
	Family effect	−4.341 (3.939)	−4.262 (3.897)
	Actor T	−0.543 (3.415)	−0.591 (2.803)
	Partner T	−17.587 (18.092)	−17.375 (14.718)
**Physical**			
	Family effect	−0.973 (2.279)	−1.028 (2.262)
	Actor T	−0.780 (2.123)	−0.791 (1.627)
	Partner T	−12.391 (11.399)	−12.100 (8.543)

^*^p < 0.05, ^**^ p < 0.01, and ^***^p < 0.001.

No significant effects are found on relational and physical aggression at wave 3. These results are not in line with previous studies, since perceived support in general has been linked to have a negative effect on internalizing and externalizing problem behaviors of the adolescent (e.g., Barrera et al., 1993; Fuhrman and Holmbeck, 1995; Scholte et al., 2001; Demaray and Malecki, 2002; Branje et al., 2004; Benhorin and McMahon, 2008). Interestingly, no evidence was found that the supportive familial environment is associated with adolescent adjustment. This is in contrast to the results of the naive ANOVA analysis presented before (Figure 2). The latter approach was, however, found to be biased in the simulation study.

### 6.2. SRM-components of perceived support as outcome

Second, we consider several antecedents of the SRM-components (see Figure 3). Based on the simulation study, we concluded that only SEM FIML can be used there. A SEM was fitted where the SRM-effects of perceived support at wave 2 are regressed on gender of the target adolescent, victimhood of physical and relational aggression at wave 1. This model fits well [$\chi_{\left(62\right)}^{2}=99.597$ *p* = 0.002, CFI = 0.979, SRMR = 0.041, RMSEA = 0.035].

The results, summarized in Table 7, reveal no impact of gender on the family effect of support nor on any of the actor effects. There is, however, an impact of gender on the partner effect of the father. That is, in families where the adolescent is a girl the father will be experienced as less supportive than in families where the adolescent is a boy. The partner effect of the father depends on the perception of support of the mother and the son or the daughter. For the first, research has shown that marital support is more important for the parent that is present in dyads of a parent and a child of the opposite sex. This means that in families where the target adolescent is a girl the mother will need less support from her partner. For the second, it has been pointed out that support is more important for parents of a son than parents of a daughter, since boys tend to spend more time outside the family than girls do (Bogenschneider et al., 1997). Finally, girls perceive in general more support from their peers than from their family, while boys perceive more support from their family than from their peers (Rueger et al., 2010). There is also a significant negative effect of perceived victimhood of relational aggression on the supportive familial environment and of perceived victimhood of physical aggression on the actor effect of the target adolescent. In families where the adolescent perceives him-or herself as a victim of relational aggression, there is on average less perceived support present within the family. A review of Meeus (2016) on longitudinal studies on adolescent psychosocial development found that adolescent psychopathology leads to lower levels of parent-adolescent relationship quality. This might explain why victimhood has a negative association with perceived familial support. However, the effect here might actually be reversed. Research on victims and bullies by Demaray and Malecki (2003) shows, for example, that victims and bullies experience less support compared to the comparison group of students. A meta-analysis confirms that positive parenting behavior, and thus more familial support, is protective against (peer) victimization (Lereya et al., 2013). Interestingly, a positive effect of perceived victimhood of relational aggression is observed on the actor effect of the mother. Also these results are partly in contrast to those of the naive ANOVA analysis (Figure 3). The discrepancy may be explained by the missing data mechanism. Indeed ANOVA-scores as outcome are only unbiased in the SRM-setting when missingness is completely at random.

**Table 7:** Casestudy: SRM-components as outcome.

	**Parameters**	**SEM FIML (s.e.)**
**Family effect**		
	Victim Rel	−0.014^***^ (0.004)
	Victim Phys	−0.006 (0.007)
	Gender	0.021 (0.031)
**Actor T**		
	Victim Rel	−0.006 (0.005)
	Victim Phys	−0.018^*^ (0.009)
	Gender	−0.038 (0.040)
**Actor M**		
	Victim Rel	0.016^**^ (0.005)
	Victim Phys	0.001 (0.008)
	Gender	0.044 (0.036)
**Actor F**		
	Victim Rel	0.001 (0.005)
	Victim Phys	0.011 (0.009)
	Gender	−0.057 (0.038)
**Actor S**		
	Victim Rel	−0.010 (0.006)
	Victim Phys	0.006 (0.010)
	Gender	0.050 (0.042)
**Partner T**		
	Victim Rel	−0.001 (0.003)
	Victim Phys	−0.005 (0.006)
	Gender	−0.020 (0.024)
**Partner M**		
	Victim Rel	0.003 (0.004)
	Victim Phys	0.005 (0.006)
	Gender	0.047 (0.027)
**Partner F**		
	Victim Rel	−0.001 (0.004)
	Victim Phys	−0.005 (0.008)
	Gender	−0.067^*^ (0.033)
**Partner S**		
	Victim Rel	−0.001 (0.004)
	Victim Phys	0.005 (0.006)
	Gender	0.040 (0.027)

* *p < 0.05*,

** p < 0.01, and

*** *p < 0.001*.

## 7. Discussion

In this article a simulation study was presented that compared the regression (FIML) and Bartlett (FIML) approaches with SEM and the ANOVA-based approach.

The simulation study showed that when the SRM-components are used as predictor, regression (FIML) FSR is a valuable alternative for SEM. There are, however, some limitations with regression (FIML) FSR. First, the standard errors of the FSR are underestimated even in large samples, but bootstrap may help overcome this limitation. Second, regression FIML has stronger missingness assumptions than SEM. Indeed, for regression FIML missingness may only depend on observed dyadic scores used in the SRM. The upside of regression FIML FSR is that it has less convergency issues than SEM in small samples.

In the second condition, where the SRM-components are used as outcome, SEM clearly outperforms all the other methods concerning bias, coverage and precision. These results are not in line with previous research that suggested the Bartlett factor scores as a valuable alternative. An explanation for this deviation was found in the use of the Moore-Penrose generalized pseudo-inverse that rendered the unbiasedness characteristic of the Bartlett factor scores void. Factor loading matrices that are not full rank may also appear in other settings, such as multitrait-multimethods models (Grayson and Marsh, 1994). The findings presented in this paper are therefore likely to be more broadly applicable than the SRM.

Our results clearly provide some challenges for future research. First, a big challenge would be to find an alternative conditionally unbiased factor score estimation method for more complex models in the presence of missing data where the factor loading matrix is not full rank. Second, if one is interested in a model where a latent variable functions as both a dependent and independent variable, another challenge occurs since none of the discussed factor scoring methods results in unbiased estimates in both conditions. Methods that fix this problem transform the Bartlett factor scores in such a way that they also result in unbiased coefficients if used as independent variable e.g., the method of Croon (Croon, 2002) and the Hoshino-Bentler method (Hoshino and Bentler, 2011). Since the Bartlett factor scores are not suitable for the SRM, these methods can not be used to investigate structures where the SRM-components are used simultaneously as outcome and predictor. Finding a suitable transformation or alternative estimation method that allows to use the SRM-components as dependent and independent variable at the same time thus remains an open research question. Third, the factor scores in popular SEM-packages such as lavaan (Rosseel, 2012) or Mplus (Muthén and Muthén, 2012) are based on the SRM variances estimated using maximum likelihood (ML). To overcome the biased ML-based variance estimates, the SRM-variances could be estimated using restricted maximum likelihood (REML) (Nestler, 2016). Future research should investigate the impact of REML estimation on the small sample performance of the different factor score estimation methods. However, to the best of our knowledge no SEM package, besides the *R*-package OpenMx (Neale et al., 2016), easily provides REML estimates.

## Author's note

In the present study, data of the RADAR-Y (Research on Adolescent Development and Relationships-Younger cohort) study were used. RADAR-Y has been financially supported by main grants from the Netherlands Organization for Scientific Research (GB-MAGW 480-03-005; GB-MAGW 480-08-006), and Stichting Achmea Slachtoffer en Samenleving (SASS), a grant from the Netherlands Organization for Scientific Research to the Consortium Individual Development (CID; 024.001.003), and various other grants from the Netherlands Organization for Scientific Research (NWO), the VU University of Amsterdam and Utrecht University. JL, AB, and TL would like to thank the Research Foundation Flanders (FWO) for financial support (Grant G020115N).

## Author contributions

JL, TL, and AB contributed to the conception and design of the manuscript. Data collection was carried out by SB and WM. Data analysis and interpretation for this study was performed by JL, TL, and VE. Next, JL developed the initial draft of the manuscript. Finally, all authors approve the version to be published.

### Conflict of interest statement

The authors declare that the research was conducted in the absence of any commercial or financial relationships that could be construed as a potential conflict of interest.

## Appendix A: derivation of the ANOVA scores

According to the SRM, each *X*_*ij*_ can be expressed as a linear function of a family effect (*Fam*), an actor effect (*Act*), a partner effect (*Par*) and measurement error (ϵ):

(A1) $$\begin{array}{r} X_{ij}=Fam+Act_{i}+Par_{j}+\epsilon_{ij} \end{array}$$

In the following we derive the ANOVA scores for the SRM effects for *Fam*, *Act*_*i*_ and *Par*_*j*_ (Equations 3 and 4 in the body of the text). When using effect coding, we have for example that *Act*_*S*_ and *Par*_*S*_ can be specified as −*Act*_*M*_−*Act*_*F*_−*Act*_*T*_ and −*Par*_*M*_−*Par*_*F*_−*Par*_*T*_, respectively. Using matrix notation the above 12 equations for *X*_*ij*_ can be rewritten as

(A2) $$\begin{array}{r} \text{X}=\text{L}\text{B}+\epsilon \end{array}$$

where **X** is a 12 × 1 matrix that contains the observed dyadic values, **L** is a 12 × 7 weight matrix, **B** is a 7 × 1 matrix with the SRM effects and ϵ is a 12 × 1 matrix that contains the measurement error. More specifically, we have the following **L** matrix:

and the following **B** matrix:

$$B=\left[\begin{array}{c} Fam\\ Act_{M}\\ Act_{F}\\ Act_{T}\\ Par_{M}\\ Par_{F}\\ Par_{T} \end{array}\right]$$

The ANOVA scores for the SRM-effects are obtained as the OLS-estimators for **B**, i.e.,

(A3) $$\begin{array}{r} \hat{\text{B}}={\left({\text{L}}^{\prime }\text{L}\right)}^{-1}{\text{L}}^{\prime }\text{X} \end{array}$$
