# Supplementary material for: Factor Score Regression With Social Relations Model Components: A Case Study Exploring Antecedents and Consequences of Perceived Support in Families
Source: Front Psychol. 2018 Sep 19;9:1699. doi: 10.3389/fpsyg.2018.01699 (PMC6157408; doi:10.3389/fpsyg.2018.01699)
Supplement: Supplementary file 1 [file Data_Sheet_1.PDF]

Supplementary material

SRM as predictor

Complete case scenario

Bias

Table 1: Median of the parameters (N=50) (Note: * indicates significant biased estimates)

SRM	parameter	truth	SEM	Regression	Bartlett	ANOVA
y (Target)	b1	10.743	10.579	10.326*	11.069*	8.285*
	b2	1.327	1.112	1.192	0.333*	0.346*
	b3	10.794	10.126	10.276	5.096*	5.122*
y (Mother)	b1	0	0.22	0.159	0.151	0.092
	b2	0	-0.338	0.047	0.056	0.042
	b3	0	-1.433	-0.167	-0.075	0.036
y (Sibling)	b1	0	0.037	-0.038	0.003	0.039
	b2	0	-0.196	0.017	0.041	0.006
	b3	0	-0.451	-0.104	-0.124	-0.087
y (Father)	b1	0	0.003	-0.019	-0.149	-0.062
	b2	0	-0.298	-0.114	-0.004	0.006
	b3	0	-0.227	-0.199	0.012	-0.038

Table 2: Median of the parameters (N=500) (Note: * indicates significant biased estimates)

SRM	parameter	truth	SEM	Regression	Bartlett	ANOVA
y (Target)	b1	10.743	10.739	10.73	11.169*	8.31*
	b2	1.327	1.306	1.345	0.404*	0.408*
	b3	10.794	10.845	10.874	4.902*	4.898*
y (Mother)	b1	0	0.027	0.033	0.009	-0.02
	b2	0	0.018	-0.012	-0.01	-0.008
	b3	0	-0.063	-0.072	-0.033	-0.016
y (Sibling)	b1	0	0.004	0.012	-0.005	-0.003
	b2	0	0.006	0.028	0.012	0.018
	b3	0	0.037	0.083	0.034	0.033
y (Father)	b1	0	-0.029	-0.004	-0.004	0.008
	b2	0	0.035	0.018	0.018	0.015
	b3	0	0.049	-0.026	-0.026	-0.033

Coverage

Table 3: Coverage of parameter confidence intervals (N=50)

SRM	parameter	SEM	Regression	Bartlett	ANOVA
y (Target)	b1	0.907	0.918	0.945	0.667
	b2	0.877	0.874	0.906	0.903
	b3	0.809	0.841	0.302	0.279
y (Mother)	b1	0.902	0.956	0.956	0.944
	b2	0.874	0.948	0.958	0.959
	b3	0.862	0.94	0.936	0.932
y (Father)	b1	0.891	0.947	0.942	0.947
	b2	0.91	0.955	0.946	0.946
	b3	0.911	0.958	0.949	0.945
y (Sibling)	b1	0.908	0.932	0.931	0.933
	b2	0.908	0.948	0.942	0.941
	b3	0.906	0.943	0.938	0.939

Table 4: Coverage of parameter confidence intervals (N=500)

SRM	parameter	SEM	Regression	Bartlett	ANOVA
y (Target)	b1	0.959	0.93	0.919	0.001
	b2	0.959	0.909	0.623	0.622
	b3	0.953	0.866	0	0
y (Mother)	b1	0.95	0.945	0.944	0.945
	b2	0.951	0.949	0.945	0.945
	b3	0.953	0.946	0.951	0.946
y (Father)	b1	0.948	0.951	0.951	0.947
	b2	0.956	0.945	0.945	0.945
	b3	0.956	0.95	0.95	0.952
y (Sibling)	b1	0.953	0.955	0.956	0.955
	b2	0.962	0.944	0.948	0.949
	b3	0.961	0.957	0.956	0.955

Median absolute deviation

Table 5: Median absolute deviation of the parameters (N=50)

SRM	parameter	SEM	Regression	Bartlett	ANOVA
y (Target)	b1	2.002	1.93	1.673	2.458
	b2	3.15	2.791	1.476	1.442
	b3	5.457	4.862	5.698	5.672
y (Mother)	b1	2.178	2.055	2.007	1.276
	b2	3.309	2.877	1.464	1.459
	b3	4.918	4.707	1.879	1.796
y (Father)	b1	2.106	2.089	1.996	1.283
	b2	2.978	3.037	1.549	1.53
	b3	4.77	4.619	1.913	1.875
y (Sibling)	b1	1.288	1.302	1.274	0.8
	b2	1.82	1.71	0.901	0.884
	b3	3.228	2.905	1.237	1.193

Table 6: Median absolute deviation of the parameters (N=500)

SRM	parameter	SEM	Regression	Bartlett	ANOVA
y (Target)	b1	0.592	0.597	0.603	2.433
	b2	0.592	0.66	0.923	0.919
	b3	1.226	1.249	5.892	5.896
y (Mother)	b1	0.615	0.638	0.609	0.392
	b2	0.682	0.696	0.456	0.455
	b3	1.108	1.15	0.551	0.553
y (Father)	b1	0.646	0.657	0.626	0.406
	b2	0.692	0.764	0.476	0.479
	b3	1.014	1.168	0.504	0.512
y (Sibling)	b1	0.412	0.424	0.409	0.268
	b2	0.387	0.455	0.286	0.287
	b3	0.627	0.721	0.332	0.332

Missingness scenario: MAR depending on another dyadic measurement

Bias

Table 7: Median of the parameters (N=50) (Note: * indicates significant biased estimates)

SRM	parameter	truth	SEM FIML	Regression FIML	Bartlett FIML	ANOVA
y (Target)	b1	10.743	10.557	10.284*	10.946	8.328*
	b2	1.327	1.365	1.278	0.215*	0.365*
	b3	10.794	10.081	10.277	5.117*	5.09*
y (Mother)	b1	0	0.189	0.219	0.134	0.074
	b2	0	-0.342	0.123	0.032	-0.011
	b3	0	-1.202	-0.215	-0.051	0.001
y (Sibling)	b1	0	0.061	-0.041	-0.012	-0.004
	b2	0	-0.203	-0.038	0.012	0.009
	b3	0	-0.366	-0.031	-0.108	-0.044
y (Father)	b1	0	-0.037	-0.155	-0.155	-0.009
	b2	0	-0.359	0.055	0.055	-0.014
	b3	0	-0.399	0.019	0.019	0.094

Table 8: Median of the parameters (N=500) (Note: * indicates significant biased estimates)

SRM	parameter	truth	SEM FIML	Regression FIML	Bartlett FIML	ANOVA
y (Target)	b1	10.743	10.81	10.787	11.129*	8.297*
	b2	1.327	1.287	1.328	0.236*	0.403*
	b3	10.794	10.859	10.87	4.94*	4.896*
y (Mother)	b1	0	0.013	0.005	0.005	-0.032
	b2	0	0.027	-0.003	-0.005	-0.021
	b3	0	-0.065	-0.074	-0.037	-0.018
y (Sibling)	b1	0	-0.008	0.004	-0.005	0.025
	b2	0	0.018	0.018	0.011	0.014
	b3	0	0.054	0.087	0.038	0.053

SRM	parameter	truth	SEM FIML	Regression FIML	Bartlett FIML	ANOVA
y (Father)	b1	0	-0.023	0.018	0.018	0
	b2	0	0.03	0.016	0.016	0.007
	b3	0	-0.01	-0.041	-0.041	0.037*

Coverage

Table 9: Coverage of parameter confidence intervals (N=50)

SRM	parameter	SEM FIML	Regression FIML	Bartlett FIML	ANOVA
y (Target)	b1	0.892	0.916	0.945	0.725
	b2	0.861	0.87	0.893	0.908
	b3	0.793	0.84	0.306	0.354
y (Mother)	b1	0.894	0.956	0.961	0.944
	b2	0.868	0.939	0.956	0.953
	b3	0.861	0.94	0.938	0.928
y (Father)	b1	0.886	0.942	0.942	0.943
	b2	0.907	0.944	0.944	0.95
	b3	0.899	0.95	0.95	0.947
y (Sibling)	b1	0.904	0.935	0.929	0.931
	b2	0.913	0.958	0.94	0.951
	b3	0.896	0.95	0.937	0.931

Table 10: Coverage of parameter confidence intervals (N=500)

SRM	parameter	SEM FIML	Regression FIML	Bartlett FIML	ANOVA
y (Target)	b1	0.96	0.931	0.922	0.007
	b2	0.962	0.905	0.494	0.678
	b3	0.95	0.867	0	0
y (Mother)	b1	0.954	0.946	0.94	0.95
	b2	0.949	0.947	0.946	0.948
	b3	0.954	0.95	0.939	0.947
y (Father)	b1	0.947	0.952	0.952	0.951
	b2	0.957	0.943	0.943	0.943
	b3	0.955	0.95	0.95	0.944
y (Sibling)	b1	0.955	0.956	0.951	0.946
	b2	0.964	0.947	0.948	0.954
	b3	0.958	0.957	0.951	0.955

Median absolute deviation

Table 11: Median absolute deviation of the parameters (N=50)

SRM	parameter	SEM FIML	Regression FIML	Bartlett FIML	ANOVA
y (Target)	b1	2.007	2.077	1.886	2.426
	b2	3.368	3.215	1.533	1.524
	b3	5.577	5.539	5.716	5.704
y (Mother)	b1	2.207	2.16	2.117	1.435

SRM	parameter	SEM FIML	Regression FIML	Bartlett FIML	ANOVA
	b2	3.327	3.243	1.571	1.533
	b3	4.667	5.141	2.017	1.938
y (Father)	b1	2.081	2.238	2.223	1.435
	b2	3.02	3.142	1.613	1.604
	b3	4.789	5.232	2.097	2.025
y (Sibling)	b1	1.342	1.471	1.463	0.937
	b2	1.812	1.893	1.006	1.004
	b3	3.31	3.29	1.26	1.277

Table 12: Median absolute deviation of the parameters (N=500)

SRM	parameter	SEM FIML	Regression FIML	Bartlett FIML	ANOVA
y (Target)	b1	0.596	0.634	0.639	2.446
	b2	0.65	0.8	0.925	0.924
	b3	1.246	1.313	5.887	5.898
y (Mother)	b1	0.63	0.74	0.722	0.452
	b2	0.687	0.728	0.47	0.465
	b3	1.15	1.237	0.582	0.582
y (Father)	b1	0.643	0.741	0.714	0.465
	b2	0.695	0.841	0.524	0.519
	b3	1.052	1.256	0.559	0.554
y (Sibling)	b1	0.418	0.475	0.463	0.289
	b2	0.385	0.498	0.311	0.307
	b3	0.667	0.879	0.385	0.372

Missingness scenario: MAR depending on the outcome

Bias

Table 13: Median of the parameters (N=50) (Note: * indicates significant biased estimates)

SRM	parameter	truth	SEM FIML	Regression FIML	Bartlett FIML	ANOVA
y (Target)	b1	10.743	10.512	10.285*	10.993*	7.388*
	b2	1.327	1.16	1.226	0.223*	0.387*
	b3	10.794	9.879	10.205	5.132*	4.556*
y (Mother)	b1	0	0.259	0.192	0.136	-0.16
	b2	0	-0.077	0.122	0.052	-0.012
	b3	0	-1.171	-0.314	-0.06	-0.015
y (Sibling)	b1	0	-0.014	-0.052	-0.021	-0.233*
	b2	0	-0.152	-0.029	0.056	0.078
	b3	0	-0.509	-0.122	-0.131	-0.037
y (Father)	b1	0	-0.093	-0.101	-0.101	-0.339*
	b2	0	-0.398	0.01	0.01	0.018
	b3	0	-0.421	0.042	0.042	0.198

Table 14: Median of the parameters (N=500) (Note: * indicates significant biased estimates)

SRM	parameter	truth	SEM FIML	Regression FIML	Bartlett FIML	ANOVA
y (Target)	b1	10.743	10.751	10.743	11.127*	7.301*
	b2	1.327	1.295	1.265	0.255*	0.36*
	b3	10.794	10.834	10.863	4.945*	4.31*
y (Mother)	b1	0	0.031	0.019	0.008	-0.199*
	b2	0	0.018	-0.012	-0.001	-0.044
	b3	0	-0.086	-0.122	-0.028	-0.046
y (Sibling)	b1	0	0.009	0.001	0.001	-0.298*
	b2	0	0.027	0.021	0.007	0.012
	b3	0	0.023	0.106	0.021	0.087*
y (Father)	b1	0	-0.004	0	0	-0.36*
	b2	0	0.044	0.022	0.022	0.034
	b3	0	0.032	-0.044*	-0.044	0.08*

Coverage

Table 15: Coverage of parameter confidence intervals (N=50)

SRM	parameter	SEM FIML	Regression FIML	Bartlett FIML	ANOVA
y (Target)	b1	0.898	0.92	0.942	0.49
	b2	0.87	0.868	0.887	0.896
	b3	0.807	0.835	0.31	0.23
y (Mother)	b1	0.893	0.955	0.956	0.943
	b2	0.884	0.94	0.962	0.952
	b3	0.873	0.934	0.946	0.937
y (Father)	b1	0.884	0.943	0.943	0.939
	b2	0.897	0.949	0.949	0.936
	b3	0.897	0.941	0.941	0.945
y (Sibling)	b1	0.888	0.936	0.933	0.926
	b2	0.904	0.952	0.944	0.949
	b3	0.882	0.942	0.938	0.939

Table 16: Coverage of parameter confidence intervals (N=500)

SRM	parameter	SEM FIML	Regression FIML	Bartlett FIML	ANOVA
y (Target)	b1	0.946	0.924	0.92	0
	b2	0.963	0.907	0.51	0.615
	b3	0.954	0.869	0	0
y (Mother)	b1	0.951	0.946	0.945	0.937
	b2	0.949	0.948	0.942	0.949
	b3	0.952	0.943	0.943	0.95
y (Father)	b1	0.954	0.95	0.95	0.906
	b2	0.954	0.953	0.953	0.939
	b3	0.956	0.955	0.955	0.938
y (Sibling)	b1	0.951	0.949	0.953	0.886
	b2	0.959	0.943	0.948	0.95
	b3	0.96	0.957	0.956	0.954

Median absolute deviation

Table 17: Median absolute deviation of the parameters (N=50)

SRM	parameter	SEM FIML	Regression FIML	Bartlett FIML	ANOVA
y (Target)	b1	1.926	2.359	1.852	3.355
	b2	3.294	2.809	1.409	1.435
	b3	5.609	5.137	6.223	6.238
y (Mother)	b1	2.243	2.23	2.27	1.384
	b2	3.196	3.1	1.568	1.569
	b3	4.735	4.95	2.111	1.951
y (Father)	b1	2.208	2.508	2.446	1.521
	b2	3.004	3.075	1.667	1.617
	b3	4.645	5.131	2.216	2.165
y (Sibling)	b1	1.304	1.521	1.497	0.958
	b2	1.871	1.853	1.009	1.001
	b3	3.337	3.301	1.285	1.289

Table 18: Median absolute deviation of the parameters (N=500)

SRM	parameter	SEM FIML	Regression FIML	Bartlett FIML	ANOVA
y (Target)	b1	0.61	1.294	0.948	3.442
	b2	0.611	0.608	0.967	0.967
	b3	1.238	1.605	6.47	6.484
y (Mother)	b1	0.621	0.719	0.686	0.461
	b2	0.666	0.728	0.488	0.488
	b3	1.09	1.252	0.581	0.578
y (Father)	b1	0.647	0.856	0.79	0.504
	b2	0.705	0.861	0.52	0.509
	b3	1.044	1.3	0.585	0.58
y (Sibling)	b1	0.423	0.544	0.54	0.353
	b2	0.395	0.501	0.322	0.318
	b3	0.65	0.774	0.367	0.359

SRM as outcome

Complete case scenario

Bias

Table 19: Median of the parameters (N=50) (Note: * indicates significant biased estimates)

SRM	parameter	truth	SEM	Regression	Bartlett	ANOVA
FE	b1	0.021	0.018	0.019	0.011	0.015
	b2	-0.006	-0.006	-0.003*	-0.005	-0.007
	b3	-0.094	-0.122	-0.102	-0.073	-0.114
AT	b1	-0.202	-0.21	-0.132*	-0.2	-0.211
	b2	-0.005	-0.005	-0.003	-0.005	-0.005

SRM	parameter	truth	SEM	Regression	Bartlett	ANOVA
	b3	1.402	1.413	0.913*	1.363	1.374
AM	b1	-0.04	-0.055	-0.022*	-0.046	-0.045
	b2	-0.038	-0.038	-0.023*	-0.037	-0.038
	b3	-0.575	-0.567	-0.369*	-0.555	-0.591
AF	b1	-0.093	-0.097	-0.054*	-0.087	-0.1
	b2	-0.005	-0.005	-0.002*	-0.003	-0.003
	b3	-0.707	-0.725	-0.448*	-0.703	-0.722
AS	b1	0.335	0.338	0.206*	0.332	0.344
	b2	0.048	0.047	0.029*	0.046	0.047
	b3	-0.12	-0.11	-0.098	-0.115	-0.087

Table 20: Median of the parameters (N=500) (Note: * indicates significant biased estimates)

SRM	parameter	truth	SEM	Regression	Bartlett	ANOVA
FE	b1	0.021	0.023	0.019	0.016	0.024
	b2	-0.006	-0.005	-0.004*	-0.004*	-0.005
	b3	-0.094	-0.094	-0.097	-0.06*	-0.09
AT	b1	-0.202	-0.198	-0.141*	-0.2	-0.201
	b2	-0.005	-0.005	-0.004*	-0.005	-0.005
	b3	1.402	1.397	0.979*	1.398	1.403
AM	b1	-0.04	-0.04	-0.022*	-0.038	-0.038
	b2	-0.038	-0.039	-0.026*	-0.039	-0.039
	b3	-0.575	-0.584	-0.408*	-0.588	-0.589
AF	b1	-0.093	-0.092	-0.059*	-0.093	-0.093
	b2	-0.005	-0.005	-0.003*	-0.005	-0.005
	b3	-0.707	-0.72	-0.485*	-0.712	-0.716
AS	b1	0.335	0.336	0.227*	0.335	0.336
	b2	0.048	0.048	0.032*	0.048	0.048
	b3	-0.12	-0.115	-0.091*	-0.113	-0.115

Coverage

Table 21: Coverage of parameter confidence intervals (N=50)

SRM	parameter	SEM	Regression	Bartlett	ANOVA
FE	b1	0.918	0.926	0.923	0.928
	b2	0.914	0.925	0.921	0.917
	b3	0.92	0.932	0.925	0.927
AT	b1	0.925	0.876	0.894	0.998
	b2	0.925	0.915	0.891	0.996
	b3	0.912	0.678	0.884	0.999
AM	b1	0.916	0.882	0.89	0.925
	b2	0.923	0.806	0.898	0.936
	b3	0.918	0.816	0.899	0.922
AF	b1	0.918	0.887	0.89	0.931
	b2	0.918	0.915	0.909	0.937
	b3	0.913	0.779	0.885	0.925
AS	b1	0.924	0.787	0.893	0.927

SRM	parameter	SEM	Regression	Bartlett	ANOVA
	b2	0.904	0.761	0.881	0.932
	b3	0.923	0.897	0.898	0.941

Table 22: Coverage of parameter confidence intervals (N=500)

SRM	parameter	SEM	Regression	Bartlett	ANOVA
FE	b1	0.948	0.953	0.953	0.95
	b2	0.944	0.937	0.937	0.945
	b3	0.949	0.947	0.94	0.947
AT	b1	0.954	0.74	0.921	0.998
	b2	0.939	0.914	0.908	0.999
	b3	0.947	0.087	0.92	0.999
AM	b1	0.962	0.93	0.932	0.964
	b2	0.959	0.523	0.932	0.967
	b3	0.943	0.628	0.927	0.948
AF	b1	0.958	0.865	0.915	0.956
	b2	0.946	0.91	0.917	0.949
	b3	0.948	0.468	0.917	0.948
AS	b1	0.966	0.388	0.933	0.961
	b2	0.954	0.386	0.918	0.951
	b3	0.95	0.91	0.914	0.948

Median absolute deviation

Table 23: Median absolute deviation of the parameters (N=50)

SRM	parameter	SEM	Regression	Bartlett	ANOVA
FE	b1	0.2	0.121	0.13	0.193
	b2	0.028	0.017	0.019	0.029
	b3	0.466	0.285	0.308	0.454
AT	b1	0.174	0.132	0.175	0.174
	b2	0.026	0.017	0.025	0.026
	b3	0.416	0.512	0.404	0.406
AM	b1	0.177	0.11	0.176	0.178
	b2	0.025	0.021	0.024	0.024
	b3	0.427	0.322	0.401	0.424
AF	b1	0.185	0.117	0.179	0.173
	b2	0.023	0.014	0.023	0.024
	b3	0.415	0.352	0.398	0.391
AS	b1	0.173	0.159	0.172	0.171
	b2	0.026	0.024	0.026	0.026
	b3	0.441	0.286	0.426	0.432

Table 24: Median absolute deviation of the parameters (N=500)

SRM	parameter	SEM	Regression	Bartlett	ANOVA
FE	b1	0.059	0.038	0.04	0.058

SRM	parameter	SEM	Regression	Bartlett	ANOVA
	b2	0.008	0.006	0.006	0.008
	b3	0.137	0.086	0.092	0.135
AT	b1	0.05	0.062	0.052	0.051
	b2	0.008	0.006	0.008	0.008
	b3	0.122	0.423	0.123	0.126
AM	b1	0.051	0.035	0.051	0.05
	b2	0.007	0.012	0.008	0.008
	b3	0.128	0.168	0.128	0.129
AF	b1	0.049	0.042	0.051	0.05
	b2	0.008	0.005	0.007	0.008
	b3	0.121	0.223	0.119	0.119
AS	b1	0.048	0.108	0.05	0.049
	b2	0.007	0.016	0.007	0.007
	b3	0.132	0.089	0.131	0.133

Missingness scenario: MAR depending on another dyadic measurement

Bias

Table 25: Median of the parameters (N=50) (Note: * indicates significant biased estimates)

SRM	parameter	truth	SEM FIML	Regression FIML	Bartlett FIML	ANOVA
FE	b1	0.021	0.014	0.021	0.013	-0.032*
	b2	-0.006	-0.006	-0.004	-0.004	-0.013*
	b3	-0.094	-0.117	-0.098	-0.074	-0.068
AT	b1	-0.202	-0.207	-0.12*	-0.194	-0.176
	b2	-0.005	-0.005	-0.002*	-0.004	-0.001
	b3	1.402	1.402	0.851*	1.339*	1.381
AM	b1	-0.04	-0.059	-0.026	-0.044	-0.029
	b2	-0.038	-0.04	-0.023*	-0.038	-0.038
	b3	-0.575	-0.566	-0.352*	-0.566	-0.568
AF	b1	-0.093	-0.089	-0.047*	-0.08	-0.077
	b2	-0.005	-0.005	-0.002*	-0.003	-0.004
	b3	-0.707	-0.697	-0.39*	-0.656	-0.719
AS	b1	0.335	0.328	0.202*	0.322	0.289*
	b2	0.048	0.046	0.029*	0.046	0.042*
	b3	-0.12	-0.104	-0.08*	-0.102	-0.1

Table 26: Median of the parameters (N=500) (Note: * indicates significant biased estimates)

SRM	parameter	truth	SEM FIML	Regression FIML	Bartlett FIML	ANOVA
FE	b1	0.021	0.023	0.021	0.017	-0.038*
	b2	-0.006	-0.006	-0.003*	-0.004*	-0.012*
	b3	-0.094	-0.091	-0.106	-0.061*	-0.063*
AT	b1	-0.202	-0.199	-0.135*	-0.197	-0.18*
	b2	-0.005	-0.005	-0.003*	-0.005	-0.002*
	b3	1.402	1.4	0.923*	1.365*	1.391

SRM	parameter	truth	SEM FIML	Regression FIML	Bartlett FIML	ANOVA
AM	b1	-0.04	-0.039	-0.027*	-0.037	-0.033
	b2	-0.038	-0.039	-0.026*	-0.039	-0.037
	b3	-0.575	-0.586	-0.401*	-0.59	-0.585
AF	b1	-0.093	-0.091	-0.058*	-0.091	-0.079*
	b2	-0.005	-0.005	-0.003*	-0.005	-0.003*
	b3	-0.707	-0.712	-0.442*	-0.688*	-0.73*
AS	b1	0.335	0.335	0.223*	0.332	0.293*
	b2	0.048	0.048	0.032*	0.048	0.043*
	b3	-0.12	-0.111	-0.081*	-0.108	-0.085*

Coverage

Table 27: Coverage of parameter confidence intervals (N=50)

SRM	parameter	SEM FIML	Regression FIML	Bartlett FIML	ANOVA
FE	b1	0.924	0.931	0.927	0.913
	b2	0.916	0.926	0.923	0.901
	b3	0.919	0.931	0.922	0.918
AT	b1	0.921	0.862	0.897	0.998
	b2	0.93	0.91	0.888	0.998
	b3	0.918	0.629	0.894	0.999
AM	b1	0.918	0.887	0.888	0.919
	b2	0.922	0.81	0.904	0.935
	b3	0.917	0.813	0.89	0.922
AF	b1	0.921	0.875	0.887	0.928
	b2	0.919	0.914	0.912	0.935
	b3	0.911	0.757	0.881	0.93
AS	b1	0.923	0.78	0.888	0.918
	b2	0.906	0.757	0.882	0.925
	b3	0.926	0.911	0.889	0.92

Table 28: Coverage of parameter confidence intervals (N=500)

SRM	parameter	SEM FIML	Regression FIML	Bartlett FIML	ANOVA
FE	b1	0.951	0.951	0.946	0.904
	b2	0.951	0.939	0.937	0.918
	b3	0.951	0.95	0.946	0.941
AT	b1	0.945	0.686	0.918	0.999
	b2	0.945	0.923	0.912	1
	b3	0.952	0.041	0.92	0.999
AM	b1	0.961	0.934	0.925	0.957
	b2	0.96	0.533	0.933	0.952
	b3	0.943	0.598	0.925	0.944
AF	b1	0.955	0.843	0.916	0.949
	b2	0.948	0.916	0.924	0.949
	b3	0.943	0.334	0.907	0.947
AS	b1	0.965	0.348	0.933	0.928
	b2	0.957	0.377	0.917	0.938
	b3	0.947	0.905	0.912	0.945

Median absolute deviation

Table 29: Median absolute deviation of the parameters (N=50)

SRM	parameter	SEM FIML	Regression FIML	Bartlett FIML	ANOVA
FE	b1	0.201	0.122	0.133	0.228
	b2	0.028	0.017	0.019	0.03
	b3	0.472	0.281	0.309	0.476
AT	b1	0.19	0.14	0.185	0.2
	b2	0.026	0.015	0.026	0.028
	b3	0.431	0.562	0.416	0.479
AM	b1	0.174	0.107	0.178	0.201
	b2	0.025	0.02	0.024	0.03
	b3	0.413	0.327	0.424	0.475
AF	b1	0.196	0.117	0.187	0.213
	b2	0.024	0.014	0.024	0.028
	b3	0.443	0.369	0.406	0.481
AS	b1	0.174	0.163	0.173	0.191
	b2	0.026	0.024	0.027	0.028
	b3	0.438	0.279	0.426	0.458

Table 30: Median absolute deviation of the parameters (N=500)

SRM	parameter	SEM FIML	Regression FIML	Bartlett FIML	ANOVA
FE	b1	0.058	0.037	0.04	0.075
	b2	0.008	0.006	0.006	0.01
	b3	0.141	0.086	0.094	0.147
AT	b1	0.052	0.068	0.055	0.065
	b2	0.008	0.005	0.008	0.009
	b3	0.129	0.479	0.129	0.14
AM	b1	0.052	0.034	0.051	0.055
	b2	0.008	0.012	0.008	0.008
	b3	0.129	0.176	0.129	0.146
AF	b1	0.053	0.042	0.054	0.061
	b2	0.007	0.005	0.007	0.008
	b3	0.122	0.265	0.12	0.139
AS	b1	0.05	0.112	0.05	0.064
	b2	0.007	0.016	0.008	0.009
	b3	0.135	0.091	0.131	0.15

Missingness scenario: MAR depending on the predictor

Bias

Table 31: Median of the parameters (N=50) (Note: * indicates significant biased estimates)

SRM	parameter	truth	SEM FIML	Regression FIML	Bartlett FIML	ANOVA
FE	b1	0.021	0.015	0.022	0.009	0.021

SRM	parameter	truth	SEM FIML	Regression FIML	Bartlett FIML	ANOVA
	b2	-0.006	-0.007	-0.003*	-0.004	-0.006
	b3	-0.094	-0.12	-0.104	-0.085	-0.057
AT	b1	-0.202	-0.216	-0.113*	-0.191	-0.2
	b2	-0.005	-0.005	-0.002*	-0.003	-0.006
	b3	1.402	1.401	0.805*	1.283*	1.357
AM	b1	-0.04	-0.037	-0.024	-0.042	-0.039
	b2	-0.038	-0.037	-0.02*	-0.036	-0.038
	b3	-0.575	-0.579	-0.314*	-0.549	-0.579
AF	b1	-0.093	-0.094	-0.057*	-0.096	-0.12
	b2	-0.005	-0.005	-0.003	-0.005	-0.005
	b3	-0.707	-0.711	-0.383*	-0.651	-0.722
AS	b1	0.335	0.333	0.197*	0.333	0.351
	b2	0.048	0.046	0.028*	0.045	0.046
	b3	-0.12	-0.128	-0.073*	-0.117	-0.112

Table 32: Median of the parameters (N=500) (Note: * indicates significant biased estimates)

SRM	parameter	truth	SEM FIML	Regression FIML	Bartlett FIML	ANOVA
FE	b1	0.021	0.02	0.026*	0.017	0.023
	b2	-0.006	-0.006	-0.002*	-0.003*	-0.005
	b3	-0.094	-0.098	-0.112*	-0.068*	-0.1
AT	b1	-0.202	-0.195	-0.115*	-0.179*	-0.196
	b2	-0.005	-0.005	-0.003*	-0.004*	-0.005
	b3	1.402	1.403	0.869*	1.342*	1.395
AM	b1	-0.04	-0.04	-0.031*	-0.044	-0.043
	b2	-0.038	-0.039	-0.024*	-0.038	-0.039
	b3	-0.575	-0.585	-0.361*	-0.56	-0.592
AF	b1	-0.093	-0.094	-0.065*	-0.097	-0.103
	b2	-0.005	-0.005	-0.004	-0.006	-0.005
	b3	-0.707	-0.711	-0.432*	-0.688*	-0.698
AS	b1	0.335	0.336	0.215*	0.331	0.341
	b2	0.048	0.049	0.031*	0.047	0.048
	b3	-0.12	-0.107	-0.071*	-0.098*	-0.116

Coverage

Table 33: Coverage of parameter confidence intervals (N=50)

SRM	parameter	SEM FIML	Regression FIML	Bartlett FIML	ANOVA
FE	b1	0.929	0.933	0.923	0.934
	b2	0.913	0.924	0.918	0.902
	b3	0.919	0.921	0.922	0.918
AT	b1	0.916	0.837	0.895	0.899
	b2	0.915	0.917	0.885	0.909
	b3	0.931	0.576	0.894	0.92
AM	b1	0.926	0.897	0.889	0.915
	b2	0.909	0.777	0.876	0.926
	b3	0.912	0.776	0.886	0.913

SRM	parameter	SEM FIML	Regression FIML	Bartlett FIML	ANOVA
AV	b1	0.915	0.887	0.879	0.9
	b2	0.925	0.915	0.906	0.927
	b3	0.91	0.74	0.884	0.908
AS	b1	0.925	0.768	0.882	0.992
	b2	0.984	0.752	0.87	0.998
	b3	0.925	0.901	0.882	0.997

Table 34: Coverage of parameter confidence intervals (N=500)

SRM	parameter	SEM FIML	Regression FIML	Bartlett FIML	ANOVA
FE	b1	0.952	0.949	0.948	0.949
	b2	0.944	0.927	0.932	0.934
	b3	0.953	0.948	0.948	0.948
AT	b1	0.944	0.558	0.907	0.949
	b2	0.949	0.912	0.909	0.949
	b3	0.954	0.013	0.899	0.95
AM	b1	0.96	0.939	0.926	0.956
	b2	0.962	0.416	0.931	0.948
	b3	0.948	0.477	0.906	0.947
AF	b1	0.957	0.889	0.926	0.95
	b2	0.945	0.921	0.916	0.958
	b3	0.947	0.288	0.904	0.947
AS	b1	0.962	0.291	0.925	0.997
	b2	0.997	0.297	0.924	1
	b3	0.946	0.898	0.901	1

Median absolute deviation

Table 35: Median absolute deviation of the parameters (N=50)

SRM	parameter	SEM FIML	Regression FIML	Bartlett FIML	ANOVA
FE	b1	0.203	0.121	0.128	0.359
	b2	0.028	0.017	0.019	0.051
	b3	0.476	0.29	0.313	0.614
AT	b1	0.199	0.139	0.193	0.34
	b2	0.026	0.015	0.026	0.045
	b3	0.478	0.61	0.449	0.584
AM	b1	0.184	0.108	0.186	0.333
	b2	0.027	0.022	0.025	0.042
	b3	0.424	0.353	0.415	0.586
AF	b1	0.193	0.119	0.186	0.317
	b2	0.024	0.014	0.023	0.043
	b3	0.422	0.384	0.425	0.58
AS	b1	0.178	0.165	0.18	0.315
	b2	0.028	0.025	0.027	0.043
	b3	0.433	0.279	0.423	0.578

Table 36: Median absolute deviation of the parameters (N=500)

SRM	parameter	SEM FIML	Regression FIML	Bartlett FIML	ANOVA
FE	b1	0.06	0.037	0.04	0.104
	b2	0.008	0.006	0.006	0.015
	b3	0.139	0.085	0.091	0.195
AT	b1	0.055	0.087	0.059	0.094
	b2	0.008	0.005	0.008	0.014
	b3	0.134	0.533	0.143	0.169
AM	b1	0.053	0.033	0.056	0.089
	b2	0.008	0.014	0.008	0.013
	b3	0.132	0.214	0.129	0.176
AF	b1	0.054	0.038	0.055	0.09
	b2	0.007	0.005	0.007	0.012
	b3	0.12	0.275	0.123	0.165
AS	b1	0.05	0.12	0.05	0.088
	b2	0.007	0.017	0.007	0.013
	b3	0.13	0.093	0.135	0.177
