# Supplementary material for: Factor Score Regression With Social Relations Model Components: A Case Study Exploring Antecedents and Consequences of Perceived Support in Families
Source: Front Psychol. 2018 Sep 19;9:1699. doi: 10.3389/fpsyg.2018.01699 (PMC6157408; doi:10.3389/fpsyg.2018.01699)
Supplement: Supplementary file 2 [file Presentation_1.PDF]

Supplementary material: R-code

```
############
# Packages #
############

library(lavaan)

###############################
# The social relations model #
###############################
# Where I_J_value is a dyadic value with I = rater and J = ratee
# T = Target, M = Mother, F = Father, S = Sibling

cfa.model <-'# Family effect:
FE =~ 1*T_M_value + 1*T_F_value + 1*T_S_value + 1*M_T_value + 1*M_F_value + 1*M_S_value +
       1*F_T_value + 1*F_M_value + 1*F_S_value + 1*S_T_value + 1*S_M_value + 1*S_F_value

# Actor effects:
A.T =~ 1*T_M_value + 1*T_F_value + 1*T_S_value
A.M =~ 1*M_T_value + 1*M_F_value + 1*M_S_value
A.F =~ 1*F_T_value + 1*F_M_value + 1*F_S_value
A.S =~ 1*S_T_value + 1*S_M_value + 1*S_F_value

# Partner effects:
P.T =~ 1*M_T_value + 1*F_T_value + 1*S_T_value
P.M =~ 1*T_M_value + 1*F_M_value + 1*S_M_value
P.F =~ 1*T_F_value + 1*M_F_value + 1*S_F_value
P.S =~ 1*T_S_value + 1*M_S_value + 1*F_S_value

# Variance labels
FE ~~ .VAR.FE*FE
A.T ~~ .VAR.A.T*A.T
A.M ~~ .VAR.A.M*A.M
A.F ~~ .VAR.A.F*A.F
A.S ~~ .VAR.A.S*A.S
P.T ~~ .VAR.P.T*P.T
P.M ~~ .VAR.P.M*P.M
P.F ~~ .VAR.P.F*P.F
P.S ~~ .VAR.P.S*P.S
T_M_value ~~ T_M_value
T_F_value ~~ T_F_value
T_S_value ~~ T_S_value
M_T_value ~~ M_T_value
M_F_value ~~ M_F_value
M_S_value ~~ M_S_value
F_T_value ~~ F_T_value
F_M_value ~~ F_M_value
F_S_value ~~ F_S_value
S_T_value ~~ S_T_value
S_M_value ~~ S_M_value
S_F_value ~~ S_F_value
```

```
# Generalized reciprocity:
A.T ~~ GR.H*P.T
A.M ~~ GR.M*P.M
A.F ~~ GR.V*P.F
A.S ~~ GR.Z*P.S

# Dyadic reciprocity
T_M_value ~~ DR.T.M*M_T_value
T_F_value ~~ DR.T.F*F_T_value
T_S_value ~~ DR.T.S*S_T_value
M_F_value ~~ DR.M.F*F_M_value
M_S_value ~~ DR.M.S*S_M_value
F_S_value ~~ DR.V.S*S_F_value

## Compute structured means
# Define labels for subsequent constraints
FE ~ .means.FE*1
A.M ~ .means.A.M*1
A.F ~ .means.A.F*1
A.S ~ .means.A.S*1
A.T ~ .means.A.T*1
P.M ~ .means.P.M*1
P.F ~ .means.P.F*1
P.S ~ .means.P.S*1
P.T ~ .means.P.T*1
T_M_value ~ .means.T_M_value*1
T_F_value ~ .means.T_F_value*1
T_S_value ~ .means.T_S_value*1
M_T_value ~ .means.M_T_value*1
M_F_value ~ .means.M_F_value*1
M_S_value ~ .means.M_S_value*1
F_T_value ~ .means.F_T_value*1
F_M_value ~ .means.F_M_value*1
F_S_value ~ .means.F_S_value*1
S_T_value ~ .means.S_T_value*1
S_M_value ~ .means.S_M_value*1
S_F_value ~ .means.S_F_value*1

# set constraints on means for identifiability
.means.A.T + .means.A.F + .means.A.M + .means.A.S == 0
.means.P.T + .means.P.F + .means.P.M + .means.P.S == 0
.means.T_M_value + .means.T_F_value + .means.T_S_value == 0
.means.M_T_value + .means.M_F_value + .means.M_S_value == 0
.means.F_T_value + .means.F_M_value + .means.F_S_value == 0
.means.S_T_value + .means.S_F_value + .means.S_M_value == 0
.means.M_T_value + .means.F_T_value + .means.S_T_value == 0
.means.T_M_value + .means.F_M_value + .means.S_M_value == 0
.means.M_S_value + .means.F_S_value + .means.T_S_value == 0
.means.M_F_value + .means.T_F_value + .means.S_F_value == 0
'
fit_cfa <- lavaan(cfa.model, data=data.outcome, missing='fiml')
# missing = 'fiml', allows the user to make use of all the observed data even
# when there are missing values present
```

```r
#####################################
# Calculation of the ANOVA scores #
#####################################

FE <- c()
A.T <- c()
A.S <- c()
A.M <- c()
A.F <- c()
P.M <- c()
P.F <- c()
P.T <- c()
P.S <- c()
for (k in 1:length(data.outcome$T_M_value)){
  # Family Effect
  FE[k]    <- rowMeans(data[k,1:12])
  # Actor effect Mother
  A.M[k] <- 3 * 3/(4*2)*(data[k,]$M_T_value + data[k,]$M_F_value + data[k,]$M_S_value)/3 +
    3/(4*2)*(data[k,]$F_M_value + data[k,]$T_M_value + data[k,]$S_M_value)/3
  - 3/2 * FE[k]
  # Actor effect Father
  A.F[k] <- 3 * 3/(4*2)*(data[k,]$F_M_value + data[k,]$F_S_value + data[k,]$F_T_value)/3 +
    3/(4*2)*(data[k,]$M_F_value + data[k,]$S_F_value + data[k,]$T_F_value)/3 - 3/2 * FE[k]
  # Actor effet Sibling
  A.S[k] <- 3 * 3/(4*2)*(data[k,]$S_M_value + data[k,]$S_F_value + data[k,]$S_T_value)/3 +
    3/(4*2)*(data[k,]$M_S_value + data[k,]$F_S_value + data[k,]$T_S_value)/3 - 3/2 * FE[k]
  # Actor effect Target
  A.T[k] <- 3 * 3/(4*2)*(data[k,]$T_M_value + data[k,]$T_F_value + data[k,]$T_S_value)/3 +
    3/(4*2)*(data[k,]$M_T_value + data[k,]$F_T_value + data[k,]$S_T_value)/3 - 3/2 * FE[k]
  # Partner effect Mother
  P.M[k] <- 3 * 3/(4*2)*(data[k,]$F_M_value + data[k,]$S_M_value + data[k,]$T_M_value)/3 +
    3/(4*2)*(data[k,]$M_F_value + data[k,]$M_S_value + data[k,]$M_T_value)/3 - 3/2 * FE[k]
  # Partner effect Father
  P.F[k] <- 3 * 3/(4*2)*(data[k,]$M_F_value + data[k,]$S_F_value + data[k,]$T_F_value)/3 +
    3/(4*2)*(data[k,]$F_M_value + data[k,]$F_S_value + data[k,]$F_T_value)/3 - 3/2 * FE[k]
  # Partner effect Sibling
  P.S[k] <- 3 * 3/(4*2)*(data[k,]$M_S_value + data[k,]$F_S_value + data[k,]$T_S_value)/3 +
    3/(4*2)*(data[k,]$S_M_value + data[k,]$S_F_value + data[k,]$S_T_value)/3 - 3/2 * FE[k]
  # Partner effect Target
  P.T[k] <- 3 * 3/(4*2)*(data[k,]$M_T_value + data[k,]$F_T_value + data[k,]$S_T_value)/3 +
    3/(4*2)*(data[k,]$T_M_value + data[k,]$T_F_value + data[k,]$T_S_value)/3 - 3/2 * FE[k]
}

##################
# SRM as outcome #
##################

## Data-generating model ##
model.data.outcome <- '
# Family effect:
FE =~ 1*T_M_value + 1*T_F_value + 1*T_S_value + 1*M_T_value + 1*M_F_value + 1*M_S_value +
      1*F_T_value + 1*F_M_value + 1*F_S_value + 1*S_T_value + 1*S_M_value + 1*S_F_value
```

```
# Actor effects:
A.T =~ 1*T_M_value + 1*T_F_value + 1*T_S_value
A.M =~ 1*M_T_value + 1*M_F_value + 1*M_S_value
A.F =~ 1*F_T_value + 1*F_M_value + 1*F_S_value
A.S =~ 1*S_T_value + 1*S_M_value + 1*S_F_value

# Partner effects:
P.T =~ 1*M_T_value + 1*F_T_value + 1*S_T_value
P.M =~ 1*T_M_value + 1*F_M_value + 1*S_M_value
P.F =~ 1*T_F_value + 1*M_F_value + 1*S_F_value
P.S =~ 1*T_S_value + 1*M_S_value + 1*F_S_value

# Relationship effects:
R.T.M =~ 1* T_M_value
R.T.F =~ 1* T_F_value
R.T.S =~ 1* T_S_value
R.M.T =~ 1* M_T_value
R.M.F =~ 1* M_F_value
R.M.S =~ 1* M_S_value
R.V.T =~ 1* F_T_value
R.V.M =~ 1* F_M_value
R.V.S =~ 1* F_S_value
R.S.T =~ 1* S_T_value
R.S.M =~ 1* S_M_value
R.S.F =~ 1* S_F_value

# regression
FE ~   (0.021)*Victimhood_rel  + (-0.006)*Victimhood_phys  + (-0.094)*Gender
A.F ~  (-0.093)*Victimhood_rel + (-0.005)*Victimhood_phys  + (-0.707)*Gender
A.M ~  (-0.040)*Victimhood_rel + (-0.038)*Victimhood_phys  + (-0.575)*Gender
A.S ~  0.335*Victimhood_rel    + 0.048*Victimhood_phys     + (-0.120)*Gender
A.T ~  (-0.202)*Victimhood_rel + (-0.005)*Victimhood_phys  + 1.402*Gender


# Variance labels
FE  ~~ 1*FE
A.T ~~ 1*A.T
A.M ~~ 1*A.M
A.F ~~ 1*A.F
A.S ~~ 1*A.S
P.T ~~ 0.5*P.T
P.M ~~ 0.5*P.M
P.F ~~ 0.5*P.F
P.S ~~ 0.5*P.S

R.T.S ~~ 1.5*R.T.S
R.T.F ~~ 1.5*R.T.F
R.T.M ~~ 1.5*R.T.M
R.V.M ~~ 1.5*R.V.M
R.V.S ~~ 1.5*R.V.S
R.V.T ~~ 1.5*R.V.T
R.M.S ~~ 1.5*R.M.S
R.M.T ~~ 1.5*R.M.T
```

```
R.M.F ~~ 1.5*R.M.F
R.S.M ~~ 1.5*R.S.M
R.S.F ~~ 1.5*R.S.F
R.S.T ~~ 1.5*R.S.T


# Generalized reciprocity:
A.T ~~ 0.05*P.T
A.M ~~ 0.05*P.M
A.F ~~ (-0.05)*P.F
A.S ~~ (-0.05)*P.S

# Dyadic reciprocity:
R.T.M ~~ 0.020*R.M.T
R.T.F ~~ 0.020*R.V.T
R.T.S ~~ 0.020*R.S.T
R.M.F ~~ 0.020*R.V.M
R.M.S ~~ 0.020*R.S.M
R.V.S ~~ 0.020*R.S.F

## Compute structured means
# # Define labels for subsequent constraints
FE ~ 4.385*1
A.T ~ 1.306*1
A.M ~  2.040*1
A.F ~ 0.650*1
A.S ~ -3.996*1
P.T ~ -1.198*1
P.M ~ 0.011*1
P.F ~ 1.238*1
P.S ~ -0.051*1
R.T.M ~ -0.136*1
R.T.F ~ 0.319*1
R.T.S ~ -0.182*1
R.M.T ~ 0.031*1
R.M.F ~ -0.162*1
R.M.S ~ 0.131*1
R.V.T ~ -0.036*1
R.V.M ~ -0.016*1
R.V.S ~ 0.052*1
R.S.T ~ 0.005*1
R.S.M ~ 0.152*1
R.S.F ~ -0.157*1

T_M_value ~~ 0*T_M_value
T_F_value ~~ 0*T_F_value
T_S_value ~~ 0*T_S_value
M_T_value ~~ 0*M_T_value
M_F_value ~~ 0*M_F_value
M_S_value ~~ 0*M_S_value
F_T_value ~~ 0*F_T_value
F_M_value ~~ 0*F_M_value
F_S_value ~~ 0*F_S_value
```

```
S_T_value ~~ 0*S_T_value
S_M_value ~~ 0*S_M_value
S_F_value ~~ 0*S_F_value

# set summarys of observed variables to zero
T_M_value ~ 0*1
T_F_value ~ 0*1
T_S_value ~ 0*1
M_T_value ~ 0*1
M_F_value ~ 0*1
M_S_value ~ 0*1
F_M_value ~ 0*1
F_T_value ~ 0*1
F_S_value ~ 0*1
S_M_value ~ 0*1
S_F_value ~ 0*1
S_T_value ~ 0*1

# exogeneous variables
Victimhood_rel ~ 2.53*1
Victimhood_phys ~ 46.47*1
Gender ~ 0.895*1
Victimhood_rel ~~ 0.50*Victimhood_rel
Victimhood_phys ~~ 27.11*Victimhood_phys
Gender ~~ 0.096*Gender
Victimhood_rel ~~ (-1.0752)*Victimhood_phys
Victimhood_rel ~~ (-0.0508)*Gender
Victimhood_phys ~~ (-0.6099)*Gender'

## Generating data ##
# Number of families
N <- 500 # 50 - 500
data.outcome <- simulateData(model.data.outcome, sample.nobs=N)

# Creating missingness
# Missing completly at random (MCAR)
id_5 <- seq(1:500)
# Mising at random (MAR) dependent on dyadic measurement (outcome)
id_5 <- which(data.outcome$S_M_value < summary(data.outcome$S_M_value)[[2]])
# MAR dependent on predictor
id_5 <- which(data.outcome$Victimhood_rel < summary(data.outcome$Victimhood_rel)[[2]])
id_6 <- which(data.outcome$Victimhood_phys < summary(data.outcome$Victimhood_phys)[[2]])

# Inserting missingness
missing.rate <- .40
data.outcome$T_M_value[sample(id_5, length(id_5)*missing.rate, replace=FALSE) ] <- NA
data.outcome$T_F_value[sample(id_5, length(id_5)*missing.rate, replace=FALSE) ] <- NA
data.outcome$T_S_value[sample(id_5, length(id_5)*missing.rate, replace=FALSE) ] <- NA
data.outcome$F_M_value[sample(id_5, length(id_5)*missing.rate, replace=FALSE) ] <- NA
data.outcome$F_T_value[sample(id_5, length(id_5)*missing.rate, replace=FALSE) ] <- NA
data.outcome$F_S_value[sample(id_5, length(id_5)*missing.rate, replace=FALSE) ] <- NA

data.outcome$T_S_value[sample(id_6, length(id_6)*missing.rate, replace=FALSE) ] <- NA
```

```
data.outcome$T_M_value[sample(id_6, length(id_6)*missing.rate, replace=FALSE) ] <- NA
data.outcome$T_F_value[sample(id_6, length(id_6)*missing.rate, replace=FALSE) ] <- NA
data.outcome$M_S_value[sample(id_6, length(id_6)*missing.rate, replace=FALSE) ] <- NA
data.outcome$M_T_value[sample(id_6, length(id_6)*missing.rate, replace=FALSE) ] <- NA
data.outcome$M_F_value[sample(id_6, length(id_6)*missing.rate, replace=FALSE) ] <- NA

## SEM Model (SRM + regressions) ##
sem.model.outcome <-'
# Family effect:
FE =~ 1*T_M_value + 1*T_F_value + 1*T_S_value + 1*M_T_value + 1*M_F_value + 1*M_S_value +
      1*F_T_value + 1*F_M_value + 1*F_S_value + 1*S_T_value + 1*S_M_value + 1*S_F_value

# Actor effects:
A.T =~ 1*T_M_value + 1*T_F_value + 1*T_S_value
A.M =~ 1*M_T_value + 1*M_F_value + 1*M_S_value
A.F =~ 1*F_T_value + 1*F_M_value + 1*F_S_value
A.S =~ 1*S_T_value + 1*S_M_value + 1*S_F_value

# Partner effects:
P.T =~ 1*M_T_value + 1*F_T_value + 1*S_T_value
P.M =~ 1*T_M_value + 1*F_M_value + 1*S_M_value
P.F =~ 1*T_F_value + 1*M_F_value + 1*S_F_value
P.S =~ 1*T_S_value + 1*M_S_value + 1*F_S_value

# Relationship effects:
R.T.M =~ 1* T_M_value
R.T.F =~ 1* T_F_value
R.T.S =~ 1* T_S_value
R.M.T =~ 1* M_T_value
R.M.F =~ 1* M_F_value
R.M.S =~ 1* M_S_value
R.V.T =~ 1* F_T_value
R.V.M =~ 1* F_M_value
R.V.S =~ 1* F_S_value
R.S.T =~ 1* S_T_value
R.S.M =~ 1* S_M_value
R.S.F =~ 1* S_F_value

# regression
FE  ~  Victimhood_rel        +Victimhood_phys         + Gender
A.F ~ beta1V*Victimhood_rel  +beta2V*Victimhood_phys  + beta4V*Gender
A.M ~ beta1M*Victimhood_rel  +beta2M*Victimhood_phys  + beta4M*Gender
A.S ~ beta1Z*Victimhood_rel  +beta2Z*Victimhood_phys  + beta4Z*Gender
A.T ~ beta1H*Victimhood_rel  +beta2H*Victimhood_phys  + beta4H*Gender

# Variance labels
FE ~~ .VAR.FE*FE
A.T ~~ .VAR.A.T*A.T
A.M ~~ .VAR.A.M*A.M
A.F ~~ .VAR.A.F*A.F
A.S ~~ .VAR.A.S*A.S
P.T ~~ .VAR.P.T*P.T
P.M ~~ .VAR.P.M*P.M
```

```
P.F ~~ .VAR.P.F*P.F
P.S ~~ .VAR.P.S*P.S
R.T.M ~~ .VAR.R.T.M*R.T.M
R.T.F ~~ .VAR.R.T.F*R.T.F
R.T.S ~~ .VAR.R.T.S*R.T.S
R.M.T ~~ .VAR.R.M.T*R.M.T
R.M.F ~~ .VAR.R.M.F*R.M.F
R.M.S ~~ .VAR.R.M.S*R.M.S
R.V.T ~~ .VAR.R.V.T*R.V.T
R.V.M ~~ .VAR.R.V.M*R.V.M
R.V.S ~~ .VAR.R.V.S*R.V.S
R.S.T ~~ .VAR.R.S.T*R.S.T
R.S.M ~~ .VAR.R.S.M*R.S.M
R.S.F ~~ .VAR.R.S.F*R.S.F

# Generalized reciprocity:
A.T ~~ GR.H*P.T
A.M ~~ GR.M*P.M
A.F ~~ GR.V*P.F
A.S ~~ GR.Z*P.S

# Dyadic reciprocity:
R.T.M ~~ DR.T.M*R.M.T
R.T.F ~~ DR.T.F*R.V.T
R.T.S ~~ DR.T.S*R.S.T
R.M.F ~~ DR.M.F*R.V.M
R.M.S ~~ DR.M.S*R.S.M
R.V.S ~~ DR.V.S*R.S.F

T_M_value ~~ 0*T_M_value
T_F_value ~~ 0*T_F_value
T_S_value ~~ 0*T_S_value
M_T_value ~~ 0*M_T_value
M_F_value ~~ 0*M_F_value
M_S_value ~~ 0*M_S_value
F_T_value ~~ 0*F_T_value
F_M_value ~~ 0*F_M_value
F_S_value ~~ 0*F_S_value
S_T_value ~~ 0*S_T_value
S_M_value ~~ 0*S_M_value
S_F_value ~~ 0*S_F_value

## Compute structured means
# Define labels for subsequent constraints
FE ~ .means.FE*1
A.T ~ .means.A.T*1
A.M ~ .means.A.M*1
A.F ~ .means.A.F*1
A.S ~ .means.A.S*1
P.T ~ .means.P.T*1
P.M ~ .means.P.M*1
P.F ~ .means.P.F*1
P.S ~ .means.P.S*1
```

```
R.T.M ~ .means.R.T.M*1
R.T.F ~ .means.R.T.F*1
R.T.S ~ .means.R.T.S*1
R.M.T ~ .means.R.M.T*1
R.M.F ~ .means.R.M.F*1
R.M.S ~ .means.R.M.S*1
R.V.T ~ .means.R.V.T*1
R.V.M ~ .means.R.V.M*1
R.V.S ~ .means.R.V.S*1
R.S.T ~ .means.R.S.T*1
R.S.M ~ .means.R.S.M*1
R.S.F ~ .means.R.S.F*1

# set means of observed variables to zero
T_M_value ~ 0*1
T_F_value ~ 0*1
T_S_value ~ 0*1
M_T_value ~ 0*1
M_F_value ~ 0*1
M_S_value ~ 0*1
F_T_value ~ 0*1
F_M_value ~ 0*1
F_S_value ~ 0*1
S_T_value ~ 0*1
S_M_value ~ 0*1
S_F_value ~ 0*1

# set constraints on means for identifiability
.means.A.T + .means.A.M + .means.A.F + .means.A.S == 0
.means.P.T + .means.P.M + .means.P.F + .means.P.S == 0
.means.R.T.M + .means.R.T.F + .means.R.T.S == 0
.means.R.M.T + .means.R.M.F + .means.R.M.S == 0
.means.R.V.T + .means.R.V.M + .means.R.V.S == 0
.means.R.S.T + .means.R.S.M + .means.R.S.F == 0
.means.R.M.T + .means.R.V.T + .means.R.S.T == 0
.means.R.T.M + .means.R.V.M + .means.R.S.M == 0
.means.R.T.F + .means.R.M.F + .means.R.S.F == 0
.means.R.T.S + .means.R.M.S + .means.R.V.S == 0

# set constraints on regression coefficients
# conditional on the predictor the means must sum to zero
beta1V + beta1M + beta1Z + beta1H == 0
beta2V + beta2M + beta2Z + beta2H == 0
beta4V + beta4M + beta4Z + beta4H == 0'
fit_sem_fiml <- lavaan(sem.model.outcome, data=data.outcome, missing='fiml')
fit_sem_cc <- lavaan(sem.model.outcome, data=data.outcome)

## CFA Model (which is th SRM model) ##
# See above "cfa.model"
fit_cfa <- lavaan(cfa.model, data=data.outcome, missing='fiml')

## Path Models ##
path_actor <- '
```

```
A.F ~ means.AV*1 + beta1V*Victimhood_rel  +beta2V*Victimhood_phys  + beta4V*Gender
A.M ~ means.AM*1 + beta1M*Victimhood_rel  +beta2M*Victimhood_phys  + beta4M*Gender
A.S ~ means.AZ*1 + beta1Z*Victimhood_rel  +beta2Z*Victimhood_phys  + beta4Z*Gender
A.T ~ means.AH*1 + beta1H*Victimhood_rel  +beta2H*Victimhood_phys  + beta4H*Gender

Victimhood_rel ~~ Victimhood_phys + Gender
Victimhood_phys ~~ Gender

A.M ~~ 0*A.S + 0*A.F + 0*A.T
A.S ~~ 0*A.F + 0*A.T
A.F ~~ 0*A.T

means.AH == 0 - means.AM - means.AZ - means.AV
beta1H == 0 - beta1M - beta1Z - beta1V
beta2H == 0 - beta2M - beta2Z - beta2V
beta4H == 0 - beta4M - beta4Z - beta4V
'
# Alternative specification
path_actor <- '
A.F ~ means.AV*1 + beta1V*Victimhood_rel  +beta2V*Victimhood_phys  + beta4V*Gender
A.M ~ means.AM*1 + beta1M*Victimhood_rel  +beta2M*Victimhood_phys  + beta4M*Gender
A.S ~ means.AZ*1 + beta1Z*Victimhood_rel  +beta2Z*Victimhood_phys  + beta4Z*Gender

Victimhood_rel ~~ Victimhood_phys + Gender
Victimhood_phys ~~ Gender

A.M ~~ 0*A.S + 0*A.F
A.S ~~ 0*A.F

means.AH := 0 - means.AM - means.AZ - means.AV
beta1H := 0 - beta1M - beta1Z - beta1V
beta2H := 0 - beta2M - beta2Z - beta2V
beta4H := 0 - beta4M - beta4Z - beta4V
'

path_FE <-'
FE  ~  means.FE*1 + Victimhood_rel        + Victimhood_phys         + Gender   '

path_partner <- '
P.F ~ means.PV*1 + beta1V*Victimhood_rel  +beta2V*Victimhood_phys  + beta4V*Gender
P.M ~ means.PM*1 + beta1M*Victimhood_rel  +beta2M*Victimhood_phys  + beta4M*Gender
P.S ~ means.PZ*1 + beta1Z*Victimhood_rel  +beta2Z*Victimhood_phys  + beta4Z*Gender
P.T ~ means.PH*1 + beta1H*Victimhood_rel  +beta2H*Victimhood_phys  + beta4H*Gender

Victimhood_rel ~~ Victimhood_phys + Gender
Victimhood_phys ~~ Gender

P.M ~~ 0*P.S + 0*P.F + 0*P.T
P.S ~~ 0*P.F + 0*P.T
P.F ~~ 0*P.T

means.PH == 0 - means.PM - means.PZ - means.PV
beta1H == 0 - beta1M - beta1Z - beta1V
```

```
beta2H == 0 - beta2M - beta2Z - beta2V
beta4H == 0 - beta4M - beta4Z - beta4V
'

## Calculating factor scores ##
## Obtain parameters of SRM-model
# Residual covariance matrix
THETA <- lavaan:::computeTHETA(lavmodel=fit_cfa@Model)[[1]]
# Latent variable covariance matrix
VETA <- lavaan:::computeVETA(lavmodel = fit_cfa@Model)[[1]]
# Means of latent variables
EETA <- lavaan:::computeEETA(lavmodel = fit_cfa@Model,
                             lavsamplestats = fit_cfa@SampleStats)[[1]]
# Factor loading matrix
LAMBDA <- lavaan:::computeLAMBDA(lavmodel = fit_cfa@Model,
                                 remove.dummy.lv = FALSE)[[1]]
# Model-implied covariance matrix
Sigma.hat <- lavaan:::computeSigmaHat(lavmodel = fit_cfa@Model)[[1]]
# Observed means of the dyadic values
EY <- unlist(lavaan:::computeEY(lavmodel=fit_cfa@Model,
                                lavsamplestats=fit_cfa@SampleStats))

## Regression FS
FS_regression <- lavPredict(fit_cfa, method='Regression')
# In specific this contains the following calculations:
# 1. Factor score coefficient of the regression factor scores
FSC_R <- VETA %*% t(LAMBDA) %*% solve(Sigma.hat)
# Alternatively one can define the FSC:
FSC_R2 <- solve(solve(VETA) +
                  t(LAMBDA)%*%solve(THETA)%*%LAMBDA) %*% t(LAMBDA) %*% solve(THETA)
# 2. Observed data
data.obs<- cbind(data.outcome$T_M_value, data.outcome$T_F_value, data.outcome$T_S_value,
                 data.outcome$M_T_value, data.outcome$M_F_value, data.outcome$M_S_value,
                 data.outcome$F_T_value, data.outcome$F_M_value, data.outcome$F_S_value,
                 data.outcome$S_T_value, data.outcome$S_M_value, data.outcome$S_F_value)

# 3. Residuals (observed data minus their mean)
RES  <- sweep(data.obs,  MARGIN = 2L, STATS = EY,   FUN = "-")
# 4. Getting rid of the missing values (ML-approach)
ML <- cbind(ifelse(is.na(data.outcome$T_M_value),0, RES[,1]),
            ifelse(is.na(data.outcome$T_F_value),0, RES[,2]),
            ifelse(is.na(data.outcome$T_S_value),0, RES[,3]),
            ifelse(is.na(data.outcome$M_T_value),0, RES[,4]),
            ifelse(is.na(data.outcome$M_F_value),0, RES[,5]),
            ifelse(is.na(data.outcome$M_S_value),0, RES[,6]),
            ifelse(is.na(data.outcome$F_T_value),0, RES[,7]),
            ifelse(is.na(data.outcome$F_M_value),0, RES[,8]),
            ifelse(is.na(data.outcome$F_S_value),0, RES[,9]),
            ifelse(is.na(data.outcome$S_T_value),0, RES[,10]),
            ifelse(is.na(data.outcome$S_M_value),0, RES[,11]),
            ifelse(is.na(data.outcome$S_F_value),0,RES[,12]))
# 5. Calculation of the Regression factor scores
# (multiplying adjusted RES with adjusted FSC and adding latent variables' means)
```

```r
FS_regression_ML <- matrix(NA, ncol=9, nrow=N)
for(i in 1:N){
  id_mis <- which(is.na(data.obs[i,]))
  if(length(id_mis) == 0){
    FSC_R <- t(solve(solve(VETA) + t(LAMBDA)%*%solve(THETA)%*%LAMBDA) %*%
                 t(LAMBDA) %*% solve(THETA))
    FS_regression_ML[i,1:9] <- (sweep(ML[i,]%*%FSC_R, MARGIN = 2L,
                                       STATS = EETA, FUN = "+"))
  } else{
    FSC_R <- t(solve(solve(VETA) + t(LAMBDA[-id_mis,])%*%
                       solve(THETA[-id_mis,-id_mis])%*%LAMBDA[-id_mis,]) %*%
                       t(LAMBDA[-id_mis,]) %*% solve(THETA[-id_mis,-id_mis]))
    FS_regression_ML[i,1:9] <- sweep(ML[i,-id_mis]%*%FSC_R, MARGIN = 2L,
                                      STATS = EETA, FUN = "+")
  }
}

## Bartlett FS
FS_Bartlett <- lavPredict(fit_cfa, method='Bartlett')
# In specific this contains the following calculations:
# 1. Factor score coefficient of the Bartlett factor scores
FSC_B <- t(MASS::ginv(t(LAMBDA)%*%solve(THETA)%*%LAMBDA) %*% t(LAMBDA) %*% solve(THETA))
# 2. Observed data
data.obs<- cbind(data.outcome$T_M_value, data.outcome$T_F_value, data.outcome$T_S_value,
                 data.outcome$M_T_value, data.outcome$M_F_value, data.outcome$M_S_value,
                 data.outcome$F_T_value, data.outcome$F_M_value, data.outcome$F_S_value,
                 data.outcome$S_T_value, data.outcome$S_M_value, data.outcome$S_F_value)
# 3. Residuals (observed data minus their mean)
RES  <- sweep(data.obs,  MARGIN = 2L, STATS = EY,   FUN = "-")
# 4. Getting rid of the missing values (ML-approach)
ML <- cbind(ifelse(is.na(data.outcome$T_M_value),0, RES[,1]),
           ifelse(is.na(data.outcome$T_F_value),0, RES[,2]),
           ifelse(is.na(data.outcome$T_S_value),0, RES[,3]),
           ifelse(is.na(data.outcome$M_T_value),0, RES[,4]),
           ifelse(is.na(data.outcome$M_F_value),0, RES[,5]),
           ifelse(is.na(data.outcome$M_S_value),0, RES[,6]),
           ifelse(is.na(data.outcome$F_T_value),0, RES[,7]),
           ifelse(is.na(data.outcome$F_M_value),0, RES[,8]),
           ifelse(is.na(data.outcome$F_S_value),0, RES[,9]),
           ifelse(is.na(data.outcome$S_T_value),0, RES[,10]),
           ifelse(is.na(data.outcome$S_M_value),0, RES[,11]),
           ifelse(is.na(data.outcome$S_F_value),0,RES[,12]))

# 5. Calculation of the Bartlett factor scores
# (multiplying adjusted RES with adjusted FSC and adding latent variables' means)
FS_Bartlett <- matrix(NA, ncol=9, nrow=N)
for(i in 1:N){
  id_mis <- which(is.na(data.obs[i,]))
  if(length(id_mis) == 0){
    FSC_B <- t(MASS::ginv(t(LAMBDA)%*%solve(THETA)%*%LAMBDA) %*%
                 t(LAMBDA) %*% solve(THETA))
    FS_Bartlett[i,1:9] <- (sweep(ML[i,]%*%FSC_B, MARGIN = 2L,
                                  STATS = EETA, FUN = "+"))
```

```
  } else{
    FSC_B <- t(MASS:::ginv(t(LAMBDA[-id_mis,])%*%solve(THETA[-id_mis,-id_mis])%*%
                  LAMBDA[-id_mis,]) %*% t(LAMBDA[-id_mis,]) %*%
                  solve(THETA[-id_mis,-id_mis]))
    FS_Bartlett[i,1:9] <- sweep(ML[i,-id_mis]%*%FSC_B, MARGIN = 2L,
                                STATS = EETA, FUN = "+")
  }
}

## FS ANOVA (see above)

## Fitting path analyses
fit_path_actor <- sem(path_actor, data=FS_regression, fixed.x=FALSE)
# FS_regression/FS_Bartlett/FS_FIML_regression/FS_ANOVA
fit_path_FE <- sem(path_FE, data=FS_regression, fixed.x=FALSE)
# FS_regression/FS_Bartlett/FS_FIML_regression/FS_ANOVA
fit_path_partner <- sem(path_partner, data=FS_regression, fixed.x=FALSE)
# FS_regression/FS_Bartlett/FS_FIML_regression/FS_ANOVA

####################
# SRM as predictor #
####################

## Data-generating model ##
model.data.predictor <- '
# Family effect:
FE =~ 1*T_M_value + 1*T_F_value + 1*T_S_value + 1*M_T_value + 1*M_F_value + 1*M_S_value +
      1*F_T_value + 1*F_M_value + 1*F_S_value + 1*S_T_value + 1*S_M_value + 1*S_F_value

# Actor effects:
A.T =~ 1*T_M_value + 1*T_F_value + 1*T_S_value
A.M =~ 1*M_T_value + 1*M_F_value + 1*M_S_value
A.F =~ 1*F_T_value + 1*F_M_value + 1*F_S_value
A.S =~ 1*S_T_value + 1*S_M_value + 1*S_F_value

# Partner effects:
P.T =~ 1*M_T_value + 1*F_T_value + 1*S_T_value
P.M =~ 1*T_M_value + 1*F_M_value + 1*S_M_value
P.F =~ 1*T_F_value + 1*M_F_value + 1*S_F_value
P.S =~ 1*T_S_value + 1*M_S_value + 1*F_S_value

# Relationship effects:
R.T.M =~ 1* T_M_value
R.T.F =~ 1* T_F_value
R.T.S =~ 1* T_S_value
R.M.T =~ 1* M_T_value
R.M.F =~ 1* M_F_value
R.M.S =~ 1* M_S_value
R.V.T =~ 1* F_T_value
R.V.M =~ 1* F_M_value
R.V.S =~ 1* F_S_value
R.S.T =~ 1* S_T_value
R.S.M =~ 1* S_M_value
```

```
R.S.F =~ 1* S_F_value

# regression
AT_relational_agr  ~ 10.743*FE + 1.327*A.T + 10.794*P.T
AM_relational_agr ~ 0*FE + 0*A.M + 0*P.M
AS_relational_agr ~ 0*FE + 0*A.S + 0*P.S
AF_relational_agr  ~ 0*FE + 0*A.F + 0*P.F


# exogeneous variabels
AT_relational_agr  ~~ 129.117*AT_relational_agr
AM_relational_agr ~~ 247.453*AM_relational_agr
AF_relational_agr  ~~ 259.669*AF_relational_agr
AS_relational_agr ~~ 102.682*AS_relational_agr
AT_relational_agr  ~~ 68.919*AF_relational_agr + 34.470*AM_relational_agr +
                      53.324*AS_relational_agr
AF_relational_agr  ~~ 83.158*AM_relational_agr + 60.030*AS_relational_agr
AM_relational_agr ~~ 50.935*AS_relational_agr

# Variance labels
FE  ~~ 1*FE
A.T ~~ 1*A.T
A.M ~~ 1*A.M
A.F ~~ 1*A.F
A.S ~~ 1*A.S
P.T ~~ 0.5*P.T
P.M ~~ 0.5*P.M
P.F ~~ 0.5*P.F
P.S ~~ 0.5*P.S

R.T.S ~~ 1.5*R.T.S
R.T.F ~~ 1.5*R.T.F
R.T.M ~~ 1.5*R.T.M
R.V.M ~~ 1.5*R.V.M
R.V.S ~~ 1.5*R.V.S
R.V.T ~~ 1.5*R.V.T
R.M.S ~~ 1.5*R.M.S
R.M.T ~~ 1.5*R.M.T
R.M.F ~~ 1.5*R.M.F
R.S.M ~~ 1.5*R.S.M
R.S.F ~~ 1.5*R.S.F
R.S.T ~~ 1.5*R.S.T

# Generalized reciprocity:
A.T ~~ 0.05*P.T
A.M ~~ 0.05*P.M
A.F ~~ (-0.05)*P.F
A.S ~~ (-0.05)*P.S

# Dyadic reciprocity:
R.T.M ~~ 0.020*R.M.T
R.T.F ~~ 0.020*R.V.T
R.T.S ~~ 0.020*R.S.T
```

```
R.M.F ~~ 0.020*R.V.M
R.M.S ~~ 0.020*R.S.M
R.V.S ~~ 0.020*R.S.F

## Compute structured means
# # Define labels for subsequent constraints
FE  ~ 3.935*1
A.T ~ 0.161*1
A.M ~ (-0.092)*1
A.F ~ 0.022*1
A.S ~ (-0.091)*1
P.T ~ (-0.131)*1
P.M ~ 0.011*1
P.F ~ 0.171*1
P.S ~ (-0.051)*1
R.T.M ~ (-0.136)*1
R.T.F ~ 0.319*1
R.T.S ~ (-0.182)*1
R.M.T ~ 0.031*1
R.M.F ~ (-0.162)*1
R.M.S ~ 0.131*1
R.V.T ~ (-0.036)*1
R.V.M ~ (-0.016)*1
R.V.S ~ 0.052*1
R.S.T ~ 0.005*1
R.S.M ~ 0.152*1
R.S.F ~ (-0.157)*1
AT_relational_agr ~ 28.316*1
AM_relational_agr ~ 18.649*1
AS_relational_agr ~ 24.693*1
AF_relational_agr ~ 19.796*1

# set variance of observed variables to zero
T_M_value ~~ 0*T_M_value
T_F_value ~~ 0*T_F_value
T_S_value ~~ 0*T_S_value
M_T_value ~~ 0*M_T_value
M_F_value ~~ 0*M_F_value
M_S_value ~~ 0*M_S_value
F_T_value ~~ 0*F_T_value
F_M_value ~~ 0*F_M_value
F_S_value ~~ 0*F_S_value
S_T_value ~~ 0*S_T_value
S_M_value ~~ 0*S_M_value
S_F_value ~~ 0*S_F_value

# set mean of observed variables to zero
T_M_value ~ 0*1
T_F_value ~ 0*1
T_S_value ~ 0*1
M_T_value ~ 0*1
M_F_value ~ 0*1
M_S_value ~ 0*1
```

```
F_M_value ~ 0*1
F_T_value ~ 0*1
F_S_value ~ 0*1
S_M_value ~ 0*1
S_F_value ~ 0*1
S_T_value ~ 0*1
'

## Generating data ##
# Number of families
N <- 50 # 50 - 500
data.predictor <- simulateData(model.data.predictor, sample.nobs=N)

# Creating missingness
# Missing completly at random (MCAR)
id_5 <- seq(1:500)
# Mising at random (MAR) dependent on dyadic measurement (outcome)
id_5 <- which(data.predictor$T_S_value < summary(data.predictor$T_S_value)[[2]])
# MAR dependent on predictor
id_5 <- which(data.predictor$AT_relational_agr <
                summary(data.predictor$AT_relational_agr)[[2]])

# Inserting missingness
missing.rate <- .25
data.predictor$T_M_value[sample(id_5, length(id_5)*missing.rate, replace=FALSE) ] <- NA
data.predictor$T_F_value[sample(id_5, length(id_5)*missing.rate, replace=FALSE) ] <- NA
data.predictor$T_S_value[sample(id_5, length(id_5)*missing.rate, replace=FALSE) ] <- NA

## SEM Model (SRM + regressions) ##
sem.model.predictor <-'
# Family effect:
FE =~ 1*T_M_value + 1*T_F_value + 1*T_S_value + 1*M_T_value + 1*M_F_value + 1*M_S_value +
      1*F_T_value + 1*F_M_value + 1*F_S_value + 1*S_T_value + 1*S_M_value + 1*S_F_value

# Actor effects:
A.A =~ 1*T_M_value + 1*T_F_value + 1*T_S_value
A.M =~ 1*M_T_value + 1*M_F_value + 1*M_S_value
A.F =~ 1*F_T_value + 1*F_M_value + 1*F_S_value
A.S =~ 1*S_T_value + 1*S_M_value + 1*S_F_value

# Partner effects:
P.A =~ 1*M_T_value + 1*F_T_value + 1*S_T_value
P.M =~ 1*T_M_value + 1*F_M_value + 1*S_M_value
P.F =~ 1*T_F_value + 1*M_F_value + 1*S_F_value
P.S =~ 1*T_S_value + 1*M_S_value + 1*F_S_value

# Relationship effects:
R.A.M =~ 1* T_M_value
R.A.F =~ 1* T_F_value
R.A.S =~ 1* T_S_value
R.M.A =~ 1* M_T_value
R.M.F =~ 1* M_F_value
R.M.S =~ 1* M_S_value
```

```
R.V.A =~ 1* F_T_value
R.V.M =~ 1* F_M_value
R.V.S =~ 1* F_S_value
R.S.A =~ 1* S_T_value
R.S.M =~ 1* S_M_value
R.S.V =~ 1* S_F_value

# regression
AT_relational_agr ~ 1 + FE + A.A  + P.A
AM_relational_agr ~ 1 + FE + A.M  + P.M
AF_relational_agr ~ 1 + FE + A.F  + P.F
AS_relational_agr ~ 1 + FE + A.S  + P.S

# Variance labels
FE ~~ .VAR.FE*FE
A.A ~~ .VAR.A.A*A.A
A.M ~~ .VAR.A.M*A.M
A.F ~~ .VAR.A.F*A.F
A.S ~~ .VAR.A.S*A.S
P.A ~~ .VAR.P.A*P.A
P.M ~~ .VAR.P.M*P.M
P.F ~~ .VAR.P.F*P.F
P.S ~~ .VAR.P.S*P.S
R.A.M ~~ .VAR.R.A.M*R.A.M
R.A.F ~~ .VAR.R.A.F*R.A.F
R.A.S ~~ .VAR.R.A.S*R.A.S
R.M.A ~~ .VAR.R.M.A*R.M.A
R.M.F ~~ .VAR.R.M.F*R.M.F
R.M.S ~~ .VAR.R.M.S*R.M.S
R.V.A ~~ .VAR.R.V.A*R.V.A
R.V.M ~~ .VAR.R.V.M*R.V.M
R.V.S ~~ .VAR.R.V.S*R.V.S
R.S.A ~~ .VAR.R.S.A*R.S.A
R.S.M ~~ .VAR.R.S.M*R.S.M
R.S.V ~~ .VAR.R.S.V*R.S.V

# Generalized reciprocity:
A.A ~~ GR.H*P.A
A.M ~~ GR.M*P.M
A.F ~~ GR.V*P.F
A.S ~~ GR.Z*P.S

# Dyadic reciprocity:
R.A.M ~~ DR.A.M*R.M.A
R.A.F ~~ DR.A.F*R.V.A
R.A.S ~~ DR.A.S*R.S.A
R.M.F ~~ DR.M.F*R.V.M
R.M.S ~~ DR.M.S*R.S.M
R.V.S ~~ DR.V.S*R.S.V

T_M_value ~~ 0*T_M_value
T_F_value ~~ 0*T_F_value
T_S_value ~~ 0*T_S_value
```

```
M_T_value ~~ 0*M_T_value
M_F_value ~~ 0*M_F_value
M_S_value ~~ 0*M_S_value
F_T_value ~~ 0*F_T_value
F_M_value ~~ 0*F_M_value
F_S_value ~~ 0*F_S_value
S_T_value ~~ 0*S_T_value
S_M_value ~~ 0*S_M_value
S_F_value ~~ 0*S_F_value

## Compute structured means
# Define labels for subsequent constraints
FE ~ .means.FE*1
A.A ~ .means.A.A*1
A.M ~ .means.A.M*1
A.F ~ .means.A.F*1
A.S ~ .means.A.S*1
P.A ~ .means.P.A*1
P.M ~ .means.P.M*1
P.F ~ .means.P.F*1
P.S ~ .means.P.S*1
R.A.M ~ .means.R.A.M*1
R.A.F ~ .means.R.A.F*1
R.A.S ~ .means.R.A.S*1
R.M.A ~ .means.R.M.A*1
R.M.F ~ .means.R.M.F*1
R.M.S ~ .means.R.M.S*1
R.V.A ~ .means.R.V.A*1
R.V.M ~ .means.R.V.M*1
R.V.S ~ .means.R.V.S*1
R.S.A ~ .means.R.S.A*1
R.S.M ~ .means.R.S.M*1
R.S.V ~ .means.R.S.V*1

# set means of observed variables to zero
T_M_value ~ 0*1
T_F_value ~ 0*1
T_S_value ~ 0*1
M_T_value ~ 0*1
M_F_value ~ 0*1
M_S_value ~ 0*1
F_T_value ~ 0*1
F_M_value ~ 0*1
F_S_value ~ 0*1
S_T_value ~ 0*1
S_M_value ~ 0*1
S_F_value ~ 0*1

# set constraints on means for identifiability
.means.A.A + .means.A.M + .means.A.F + .means.A.S == 0
.means.P.A + .means.P.M + .means.P.F + .means.P.S == 0
.means.R.A.M + .means.R.A.F + .means.R.A.S == 0
.means.R.M.A + .means.R.M.F + .means.R.M.S == 0
```

```
.means.R.V.A + .means.R.V.M + .means.R.V.S == 0
.means.R.S.A + .means.R.S.M + .means.R.S.V == 0
.means.R.M.A + .means.R.V.A + .means.R.S.A == 0
.means.R.A.M + .means.R.V.M + .means.R.S.M == 0
.means.R.A.F + .means.R.M.F + .means.R.S.V == 0
.means.R.A.S + .means.R.M.S + .means.R.V.S == 0'
fit_sem_fiml <- lavaan(sem.model.predictor, data=data.predictor, auto.var=TRUE,
                       auto.cov.y=TRUE, missing='fiml')
fit_sem_cc <- lavaan(sem.model.predictor, data=data.predictor, auto.var=TRUE,
                     auto.cov.y=TRUE)

## CFA Model (SRM model) ##
fit_cfa <- lavaan(cfa.model, data=data.predictor, missing='fiml')

## Calculation of factor scores
# see above

# linear models
fit_ratAA <- lm(AT_relational_agr ~ FE + A.A + P.A , data=FS_Regression)
# FS_regression/FS_Bartlett/FS_FIML_regression/FS_FIML_Bartlett/FS_ANOVA
fit_ratAM <- lm(AM_relational_agr ~ FE + A.M + P.M , data=FS_Regression)
# FS_regression/FS_Bartlett/FS_FIML_regression/FS_FIML_Bartlett/FS_ANOVA
fit_ratAV <- lm(AF_relational_agr ~ FE + A.F + P.F , data=FS_Regression)
# FS_regression/FS_Bartlett/FS_FIML_regression/FS_FIML_Bartlett/FS_ANOVA
fit_ratAZ <- lm(AS_relational_agr ~ FE + A.S + P.S , data=FS_Regression)
# FS_regression/FS_Bartlett/FS_FIML_regression/FS_FIML_Bartlett/FS_ANOVA
```
